# Structural basis for the indispensable role of a unique zinc finger motif in LNX2 ubiquitination

**DOI:** 10.18632/oncotarget.5326

**Published:** 2015-10-05

**Authors:** Digant Nayak, J. Sivaraman

**Affiliations:** ^1^ Department of Biological Sciences, National University of Singapore, Singapore 117543

**Keywords:** ubiquitination, Zn-finger, RING, ligand of numb, E3-ligase

## Abstract

LNX (Ligand of Numb Protein-X) proteins, LNX1 and LNX2, are RING- and PDZ-based E3-ubiquitin ligases known to interact with Numb. Silencing of LNX2 has been reported to down-regulate WNT and NOTCH, two key signaling pathways in tumorigenesis. Here we report the identification of the domain boundary of LNX2 to confer its ubiquitination activity, its crystal structure along with functional studies. We show that the RING domain in LNX2 is flanked by two Zinc-binding motifs (Zn-RING-Zn), in which the N-terminal Zinc-binding motif adopts novel conformation. Although this motif follows the typical Cys2His2-type zinc finger configuration, it is devoid of any secondary structure and forms an open circle conformation, which has not been reported yet. This unique N-terminal Zn-finger motif is indispensable for the activity and stability of LNX2, as verified using mutational studies. The Zn-RING-Zn domain of LNX2 is a dimer and assumes a rigid elongated structure that undergoes autoubiquitination and undergoes N-terminal polyubiquitination. The ubiquitin chains consist of all seven possible isopeptide linkages. These results were validated using full-length LNX2. Moreover we have demonstrated the ubiquitination of cell fate determinant protein, Numb by LNX2. Our study provides a structural basis for the functional machinery of LNX2 and thus provides the opportunity to investigate suitable drug targets against LNX2.

## INTRODUCTION

Among the numerous posttranslational modifications that exist for proteins, ubiquitination has been revealed to be an important mechanism for the spatiotemporal regulation of many cellular processes. Ubiquitination occurs through sequential steps where ubiquitin (Ub) is transferred from a ubiquitin-activating enzyme (E1) to a catalytic cysteine on the ubiquitin-conjugating enzyme (E2). The E2-Ub conjugate then cooperates with an ubiquitin ligase (E3) to transfer Ub to the substrate [1, 2] or to the E3 itself, in the case of autoubiquitination. Approximately 616 RING/U-box-dependent E3s have been found in humans [3], in contrast with just 2 known human E1 enzymes and less than 40 human E2 enzymes [4]; this large number of E3 enzymes attaches importance to their role in conferring the specificity of the ubiquitination process.

There are two predominant families of E3 ligases: HECT-type ligases [5] and RING finger-type ligases [6]. The RING-type E3 ligases are known to regulate key cellular processes, such as apoptosis [7], p53 signaling [8], and DNA repair pathways [9], among others. Abnormal regulation in these processes lead to dysregulated cell growth signals or genomic instability, which are associated with oncogenic progression [10]. It is therefore important to know how these RING-type E3 ligases regulate so many processes as well as themselves.

The LNX (Ligand of Numb protein-X) or PDZRN (PDZ and RING) family of proteins comprises five members, LNX1–LNX5 [11]. The domain architecture for these proteins shows the presence of a RING domain (Really Interesting New Gene) and one to four PDZ (PSD-95, DlgA, ZO-1) domains [11]. LNX1 protein was first identified as a binding partner to the mNumb PTB domain [12] and, later, its over-expression was shown to increase the proliferation of neuroepithelial cells rather than promoting their differentiation into neuron or glial cells [13]. Jing and co-workers [14] showed that LNX1 functions as an E3-ubiquitin ligase and degrades Numb in a ubiquitin-dependent manner. The homologous LNX2 protein also interacts with Numb and with a similar protein called Numblike [15]. LNX2 interacts with junction adhesion molecule 4 (JAM 4) [16] to augment the effect of hepatocyte growth factor (HGF) [17], an important factor in epithelial-mesenchymal transition (EMT) [18]. LNX2 is also regarded as an oncogene and found to be overexpressed in colorectal cancer patients [[19] and The Human Protein Atlas, www.proteinatlas.org/]. LNX2 activates WNT signaling, whereas silencing LNX2 reduces the expression of NOTCH1 and several of its downstream targets [19]. Moreover, LNX2 interacts with contactin-associated protein 4 (Caspr4) in a PDZ-dependent manner, a gene involved with autism spectrum disorders (ASDs) [20]. LNX2 also affects T-cell-mediated immune responses by regulating the level of the T-cell co-receptor, CD8 [21]. Yet, despite the importance of LNX2 in each of these signaling mechanisms, the structure and how it mediates its function are not yet reported.

As a continuation of our studies pertaining to E3 ligases [22–26], we sought to elucidate the functionally relevant domains of the LNX2 protein. Here, using crystal structure analysis and relevant functional studies, we report the presence of a novel zinc finger motif in LNX2 that is indispensable for its ubiquitination activity.

## RESULTS

### Identification of E2-conjugating enzyme for the autoubiquitination of LNX2

**Figure 1:**
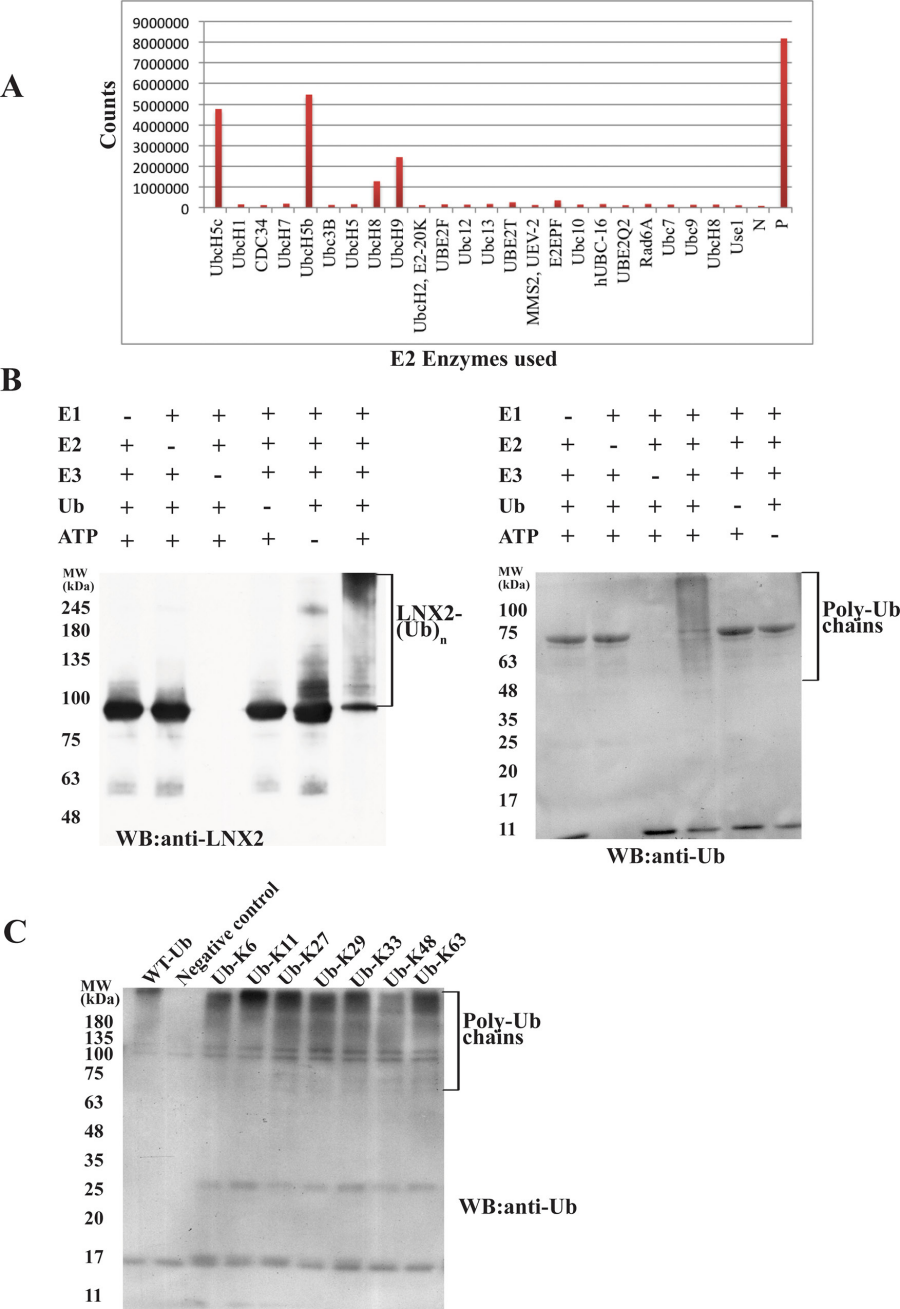
Identification of E2 for FL-LNX2 using E2 profiling and its autoubiquitination activity **A.** Result for the E2 profiling kit assay (LifeSensors). The x-axis shows all the E2 enzymes used. The *y*-axis measures the fluorescence counts. N, Negative control; P, Positive control (in the kit). **B.** *In vitro* ubiquitination assays were performed for FL-LNX2 in the presence (+) or absence (−) of each of E1, E2 (UbcH5b), FL-LNX2, Ubiquitin (Ub) and ATP respectively. Shown are the immunoblots for autoubiquitination of FL-LNX2 using anti-LNX2 antibody (left) and anti-Ubiquitin antibody (right). **C.** FL-LNX2 can form polyubiquitin chains containing all isopeptide linkages. *In vitro* ubiquitination assays were performed in the presence of E1, E2 (UbcH5b), FL-LNX2, ATP and wild-type ubiquitin or different Ub mutants, as indicated. FL-LNX2 forms polyubiquitin chains with any single-lysine mutant of ubiquitin (3^rd^ to 9^th^ lane), as shown in the immunoblot using anti-ubiquitin antibody.

Autoubiquitination is a characteristic feature of the RING-type E3 ligases [27], and serves to autoregulate the stability of the ligase [28]. We expressed the full-length LNX2 (FL-LNX2) and identified that the UbcH5 E2 family is involved in mediating the E3 ligase activity of LNX2 using the E2 profiling kit (Life Sensors) (Figure 1A). We confirmed this result using ubiquitination assays followed by western blot analysis, showing that the FL-LNX2 undergoes autoubiquitination in the presence of E1, UbcH5b E2, ubiquitin and ATP but not in the absence of UbcH5b E2 (Figure 1B).

E3-ligases tend to form specific polyubiquitin chains that comprise a single isopeptide linkage. However, Hyoung and colleagues have shown that, in the presence of UbcH5b, certain E3 ligases can form ubiquitin chains that contain all seven of the possible isopeptide linkages [29]. To identify the nature of the isopeptide linkages formed by FL-LNX2, we performed a ubiquitination assay using single lysine ubiquitin mutants, each of which containing only one lysine residue with six other lysine residues replaced by arginine. Notably, all seven ubiquitin mutants led to the formation of polyubiquitin chains (Figure 1C). To further rule out the possibility whether these ubiquitin mutants modify the types of linkages formed, we adopted the methodology used by J. Peng *et al.* ([30]). Ubiquitination assay was performed using WT-ubiquitin followed by its separation on 12.5% SDS-PAGE. The ubiquitinated species were excised, digested with trypsin and analyzed with mass spectrometry which showed the presence of distinct peptide composed of the C-terminal Gly-Gly or Lys-Arg-Gly-Gly of one ubiquitin linked to the ε-amino group of a lysine and the neighboring residues in the adjacent ubiquitin (Supplementary Figure S1). This demonstrated that FL-LNX2 formed polyubiquitin chains containing all seven possible isopeptide linkages, which is consistent with our previous results (Figure 1C).

### Polyubiquitination of human numb and effect of autoubiquitination on the substrate E3 ligase activity of LNX2

**Figure 2:**
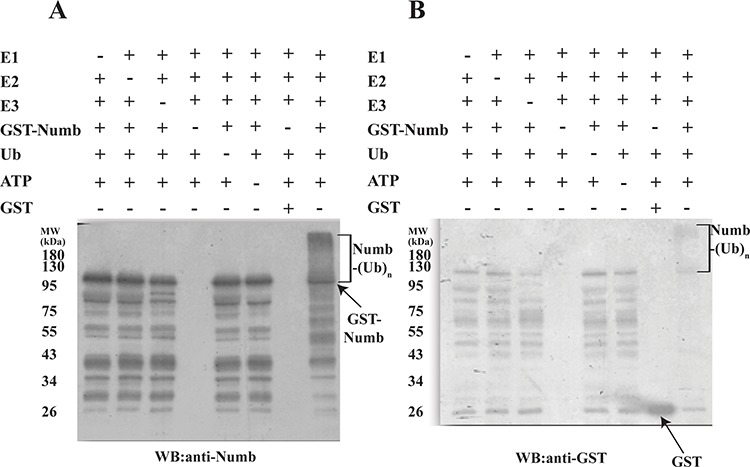
Polyubiquitination of Human Numb by LNX2 *In vitro* ubiquitination assays were performed for Numb in the presence (+) or absence (−) of each of E1, E2 (UbcH5b), FL-LNX2, GST-Numb, GST, Ubiquitin (Ub) and ATP respectively. Shown are the immunoblots for polyubiquitination of Numb using anti-Numb antibody **A.** and anti-GST antibody **B.**

Human numb plays a key role in neurogenesis and is known to bind LNX1 that leads to its proteasome-dependent degradation through ubiquitination [14, 15]. We performed an *in vitro* ubiquitination assay in the presence or absence of E1, E2, FL-LNX2, GST-Numb, Ubiquitin and ATP followed by western blot. We observed that human Numb acts as a substrate for FL-LNX2 and undergoes polyubiquitination (Figure 2A and 2B).

**Figure 3:**
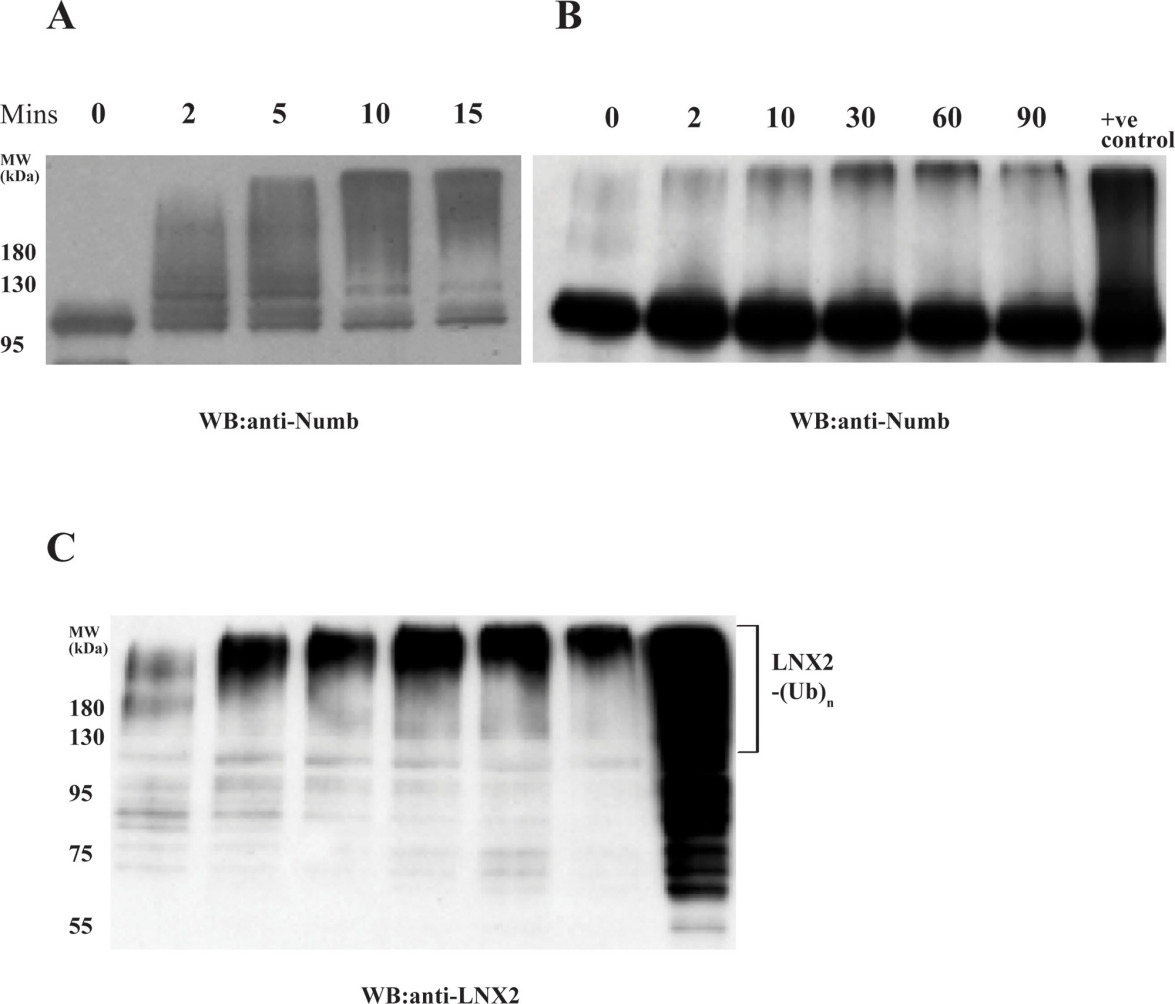
Effect of autoubiquitination of LNX2 on Numb ubiquitination *In vitro* ubiquitination assays were performed using E1, E2 (UbcH5b), unmodified FL-LNX2 or MBP beads immobilized autoubiquitinated FL-LNX2, GST-Numb, Ubiquitin and ATP at different time intervals, as indicated. +ve control-Positive control where unmodified or non-ubiquitinated FL-LNX2 was used for the ubiquitination assay. Shown are the immunoblots using anti-Numb antibodies for unmodified FL-LNX2 **A.** and autoubiquitinated FL-LNX2 **B.** The blot in (B) was stripped and again immunoblotted with anti-LNX2 antibody **C.**

Subsequently, we examined the role of autoubiquitinated LNX2 in the ubiquitination of Numb in an *in vitro* system where the autoubiquitination could be separated from the subsequent Numb ubiquitination. Maltose binding protein (MBP)- tagged FL-LNX2 was autoubiquitinated in the presence of E1, E2 and ubiquitin. After the reaction, amylose beads were used to pull down the modified or ubiquitinated FL-LNX2. The beads were washed and then used in a fresh ubiquitination reaction in the presence of Numb. Notably the autoubiquitinated LNX2 rapidly ubiquitinated the human Numb, however the ubiquitination was not as strong as compared to unmodified or non-ubiquitinated LNX2 (Figure 3A, 3B and 3C).

### Identification of ubiquitination domain and its structure

**Figure 4:**
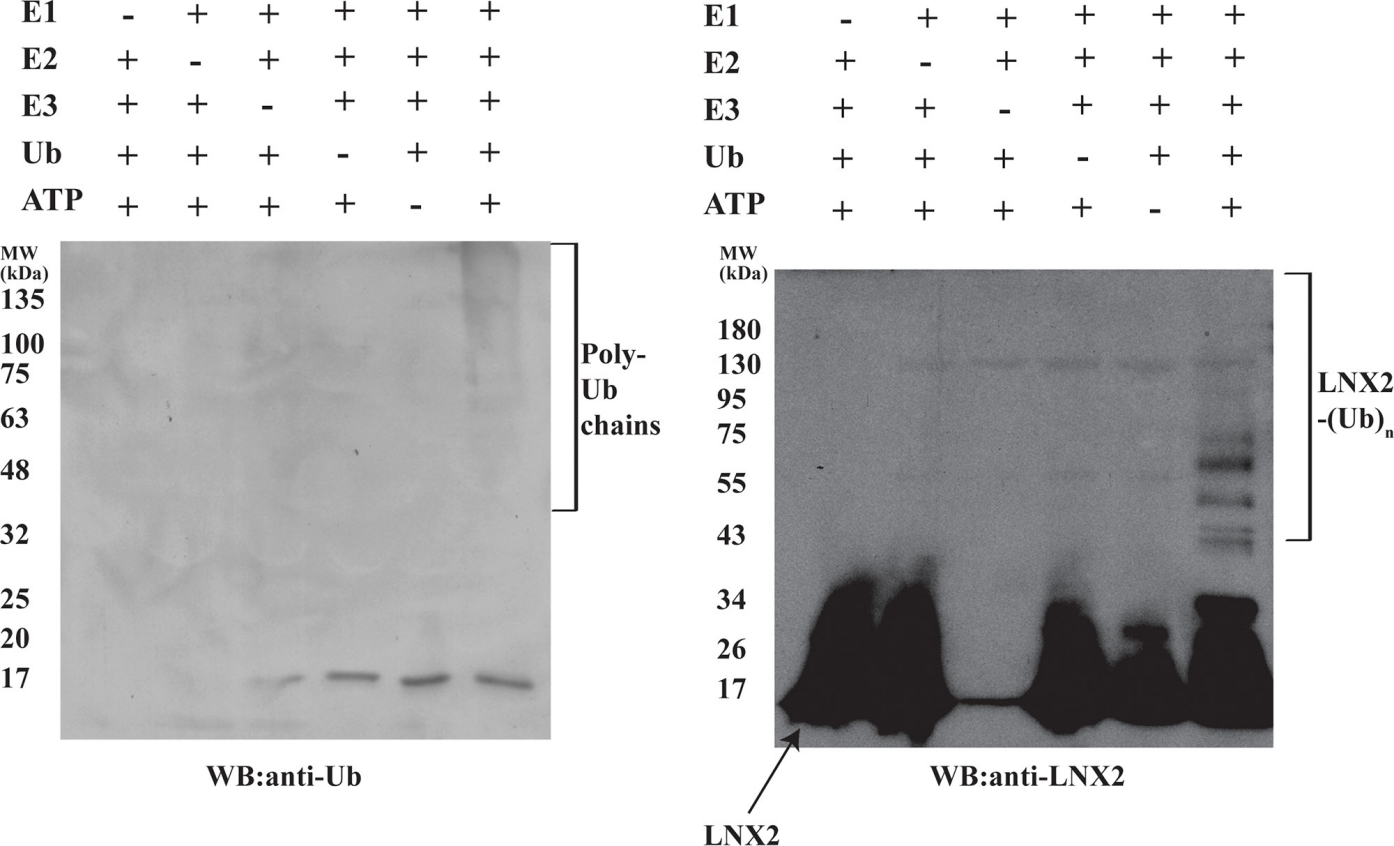
Autoubiquitination of Zn-RING-Zn domain of LNX2 *In vitro* ubiquitination assays were performed for the Zn-RING-Zn domain in the presence (+) or absence (−) of each of E1, E2 (UbcH5b), Zn-RING-Zn domain, Ubiquitin and ATP, respectively. Shown are the immunoblots for the autoubiquitination of Zn-RING-Zn domain using anti-Ubiquitin (left) and anti-LNX2 (right) antibodies.

The RING domain of RING-type E3 ligases is solely responsible for its ubiquitination activity [31]. Therefore, we sought to find the shortest sequence in FL-LNX2 that would confer ubiquitination using various truncation mutants. We found that a construct containing amino acids 20-147 of FL-LNX2 was functional and stable in solution. Repeating our ubiquitination experiments above, we found similarities between the FL-LNX2 protein and the truncated LNX2 (20-147 aa) (Figure 4).

**Figure 5:**
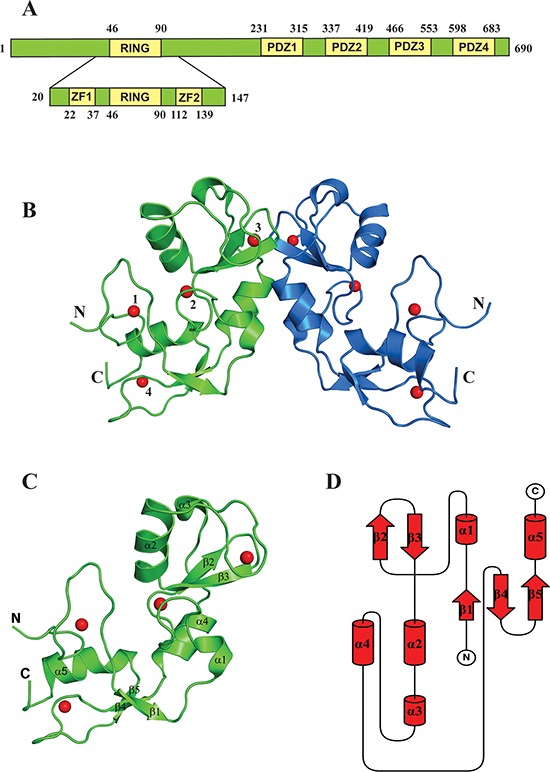
Structure of the Zn-RING-Zn domain of LNX2 **A.** Schematic representation showing the domain architecture of the full-length LNX2 (above) and the Zn-RING-Zn domain of LNX2 (below). ZF = Zinc finger domain. **B.** The crystal structure of the Zn-RING-Zn domain of LNX2 (aa 20-147). Each monomer contains four zinc coordination sites. Sites 2 and 3 are located in the RING domain. Sites 1 and 4 are coordinated by two Zinc finger motifs, respectively. **C.** Structure of the monomeric Zn-RING-Zn domain along with four coordination Zinc ions shown as red spheres. α1 and α3 are single turn helix. **D.** Topology diagram of the Zn-RING-Zn domain of LNX2, as described in (C).

Next we crystallized this construct to elucidate its structure. The structure was determined to 1.86 Å resolution (Figure 5A–5D and Supplementary Table S1). LNX2 (20-147 aa) exists as dimer in the crystal, with two molecules in the asymmetric unit (Figure 5B). The last six residues of this domain were not well defined in the electron density map and were not included in the model. The structure consists of a core RING domain (Asp46-Arg90) flanked by two zinc finger motifs on either side (hereafter referred to as the Zn-RING-Zn domain) (Figure 5A). The core RING domain of LNX2 adopts a typical RING fold, comprising one central α helix, two antiparallel β strands and two long loops. This RING domain is stabilized by two zinc ions but, unlike the Cys4HisCys3 “cross-brace” formation, one of the cysteine residues is replaced by an aspartic acid residue (Supplementary Figure S2); this has also been shown in a few other proteins, such as Rbx1 [32]. LNX2 was previously proposed to have only five domains—a N-terminal RING domain and four PDZ domains on the C-terminal [11]; the zinc finger motifs identified here were not predicted.

**Figure 6:**
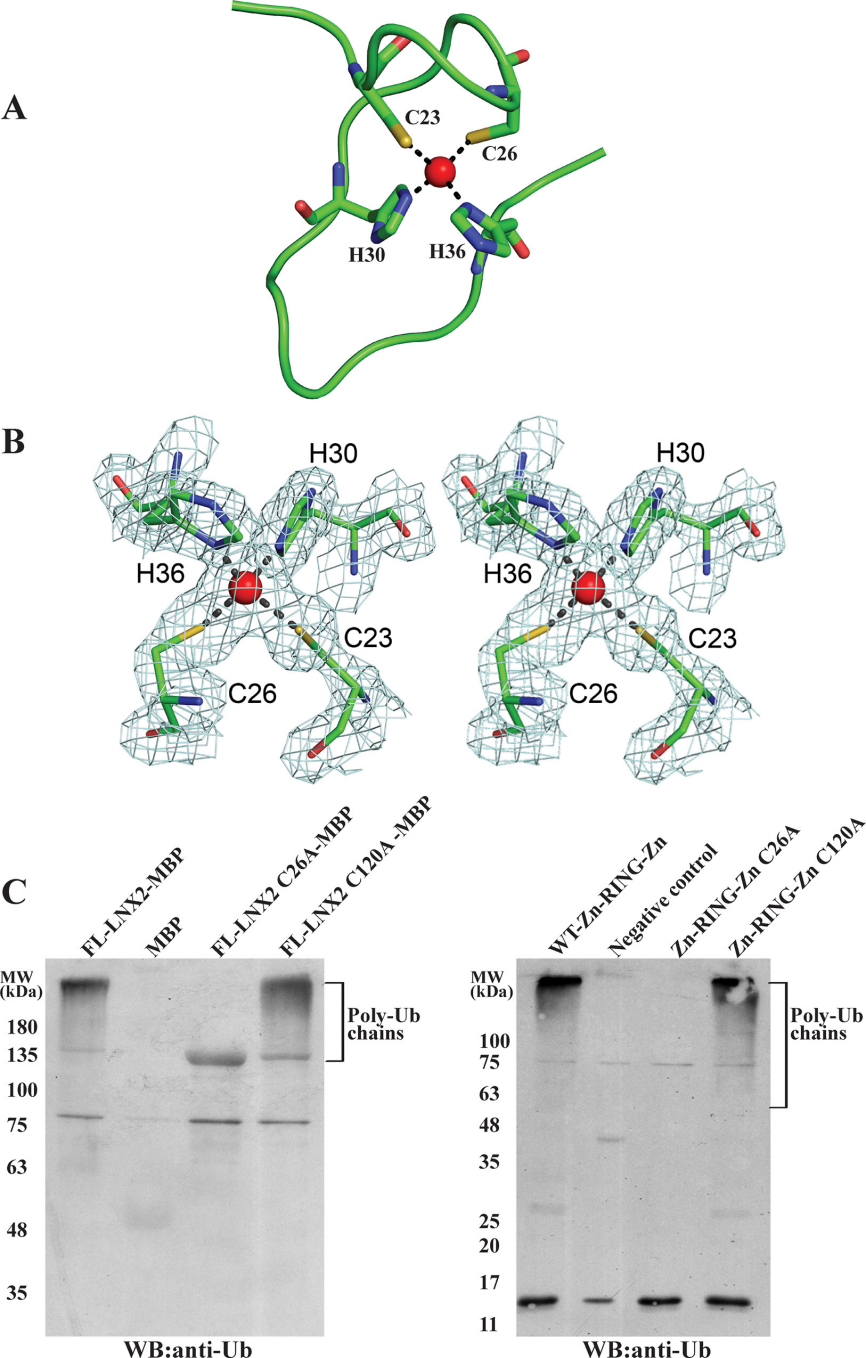
A novel Zn finger motif in the Zn-RING-Zn domain of LNX2 **A.** Structure of the N-terminal Zn finger motif in Zn-RING-Zn domain with zinc-coordinating residues and zinc ion (red sphere). **B.** The final 2F_o_-F_c_ electron density map of the N-terminal Zn finger motif contoured at 1.6 σ is shown in stereo view. The zinc-coordinating residues and the zinc ion (red sphere) are shown. **C.** Effect of mutations Cys26Ala and Cys120Ala that disrupt the N- and C- terminal Zinc finger motifs, respectively, on the activity of FL-LNX2 and Zn-RING-Zn domain. *In vitro* ubiquitination assays were performed using E1, E2 (UbcH5b), Ubiquitin, ATP and MBP-tagged mutants of FL-LNX2 (left) or mutants of Zn-RING-Zn domain (right), as indicated. Shown are the immunoblots using anti-Ubiquitin antibodies. The thick band in lane 3 from left corresponds to FL-LNX2-MBP because of the cross reactivity of anti-Ub antibody with it.

A structural homology search with the DALI server [33] (http://ekhidna.biocenter.helsinki.fi/dali_server/) identified several RING domains of other proteins, including TRAF6, that showed similarity to the 40–147aa stretch of LNX2 (i.e., the RING and C-terminal Zn finger (Supplementary Figure S3)); however, no structural homologue could be identified for the first 21 amino acids (N-terminal Zn finger) of this domain. This N-terminal Zn-finger motif forms a unique open circle conformation, with a zinc ion at the centre that stabilizes this part of the structure (Figure 6A and 6B). Although it fulfils the required criteria for a Cys2His2-type zinc finger, it does not adopt any secondary structure and does not conform to any known fold group of zinc fingers so far described. Thus, we posit that the Zn-RING-Zn domain of LNX2 is unique.

### Importance of zinc finger motifs in LNX2

The RING domain is generally considered to be sufficient to confer the ubiquitination function of RING-type E3 ligases. Next, we sought to identify the role of these two Zn finger motifs present in the Zn-RING-Zn domain. First, we truncated the beginning 21 aa of the Zn-RING-Zn domain to 47-147aa to remove the N-terminal Zn finger; however, this construct did not express in a soluble form. Next, we mutated the conserved cysteine residue to alanine in the Zn finger motifs of the Zn-RING-Zn domain to disrupt the Zn coordination. These single point mutant constructs were expressed, purified and used for ubiquitination assays. The Cys26Ala (N-terminal) mutant did not show any ubiquitination activity as compared to the wild-type Zn-RING-Zn construct and Cys120Ala (C-terminal) mutant (Figure 6C, right), and this observation was further validated using FL-LNX2 (Figure 6C, left). Collectively, these findings suggest that the N-terminal Zn finger motif is indispensable for the ubiquitination function of FL-LNX2.

### LNX2 is a dimer

**Figure 7:**
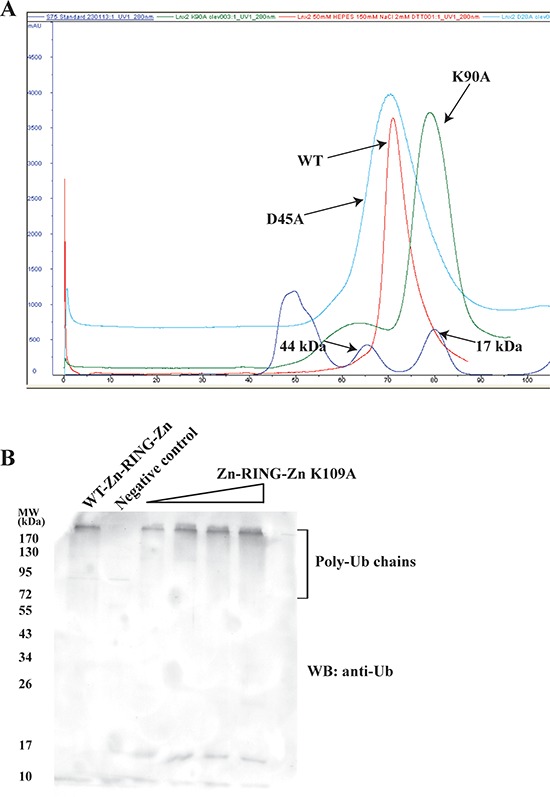
Dimer-disrupted mutant Lys109Ala and its effect on activity of Zn-RING-Zn domain **A.** WT Zn-RING-Zn and point mutants of Zn-RING-Zn, Lys109Ala and Asp45Ala were each separately used for gel-filtration chromatography. Their respective elution profiles were overlaid and compared. The molecular mass standard is shown in blue. **B.** *In vitro* ubiquitination assays were performed using E1, E2 (UbcH5b), Ubiquitin, ATP, WT-Zn-RING-Zn or Lys109Ala mutant Zn-RING-Zn with increasing concentrations, as indicated. Shown is the immunoblot using the anti-Ubiquitin antibody.

The gel filtration profiles of the Zn-RING-Zn domain and the FL-LNX2 along with the Analytical Ultracentrifugation experiment on FL-LNX2 indicate that both are dimers (Supplementary Figure S4 and S5 respectively). This is consistent with the observed Zn-RING-Zn dimers in the crystal. The dimeric interface has a buried surface area of 657.9 Å^2^ from each monomer, with six hydrogen-bonding contacts (< 3.2Å) and several hydrophobic interactions. Notably, Lys109 of one monomer makes hydrogen-bonding contacts with Asp45 and Asp47 of the other monomer in the dimeric Zn-RING-Zn domain (Supplementary Figure S6). Therefore, we next examined the role of these residues in the dimerization by point mutation analysis. The point mutants, Asp45Ala and Lys109Ala, were expressed and separated on a calibrated gel-filtration column. The gel filtration profile showed that the Lys109Ala mutant eluted as monomer (~15 kDa), whereas the Asp45Ala mutant eluted as dimer, similar to the wild-type Zn-RING-Zn domain (Figure 7A and Supplementary Figure S7B). Circular dichroism profiles for the wild type and mutant constructs showed no difference (Supplementary Figure S7A). These observations suggest that a Lys109Ala mutation disrupts the dimerization of the Zn-RING-Zn domain.

Next, we sought to understand the importance of this dimerization for LNX2 ubiquitination, using both wild-type Zn-RING-Zn and the Lys109Ala mutant in ubiquitination assays. Immunoblotting showed that both wild-type and the dimer-disrupted Lys109Ala mutant carried out equally efficient autoubiquitination (Figure 7B), suggesting that dimerization of the Zn-RING-Zn domain is not required for autoubiquitination; this is consistent with previous reports on RING-type E3 ligases [34].

To further validate our result, we sought to repeat the same experiments using FL-LNX2. According to Rice and colleagues [15], mouse LNX2 has at least two separate oligomerization sites: one at the N-terminal RING domain and the other at the extreme C-terminus (Ser685 and Val687). We mutated the N-terminal dimerization residue, Lys109A, and the corresponding C-terminal residues, Ser688 and Val690, of human FL-LNX2 to Asp and Glu, respectively, as suggested by Rice et al. This triple mutant did not elute as monomer but eluted as a higher-order oligomer in gel filtration. This might be due to the presence of other dimerization sites in FL-LNX2.

### Zn-RING-Zn domain undergoes N-terminal ubiquitination

**Figure 8:**
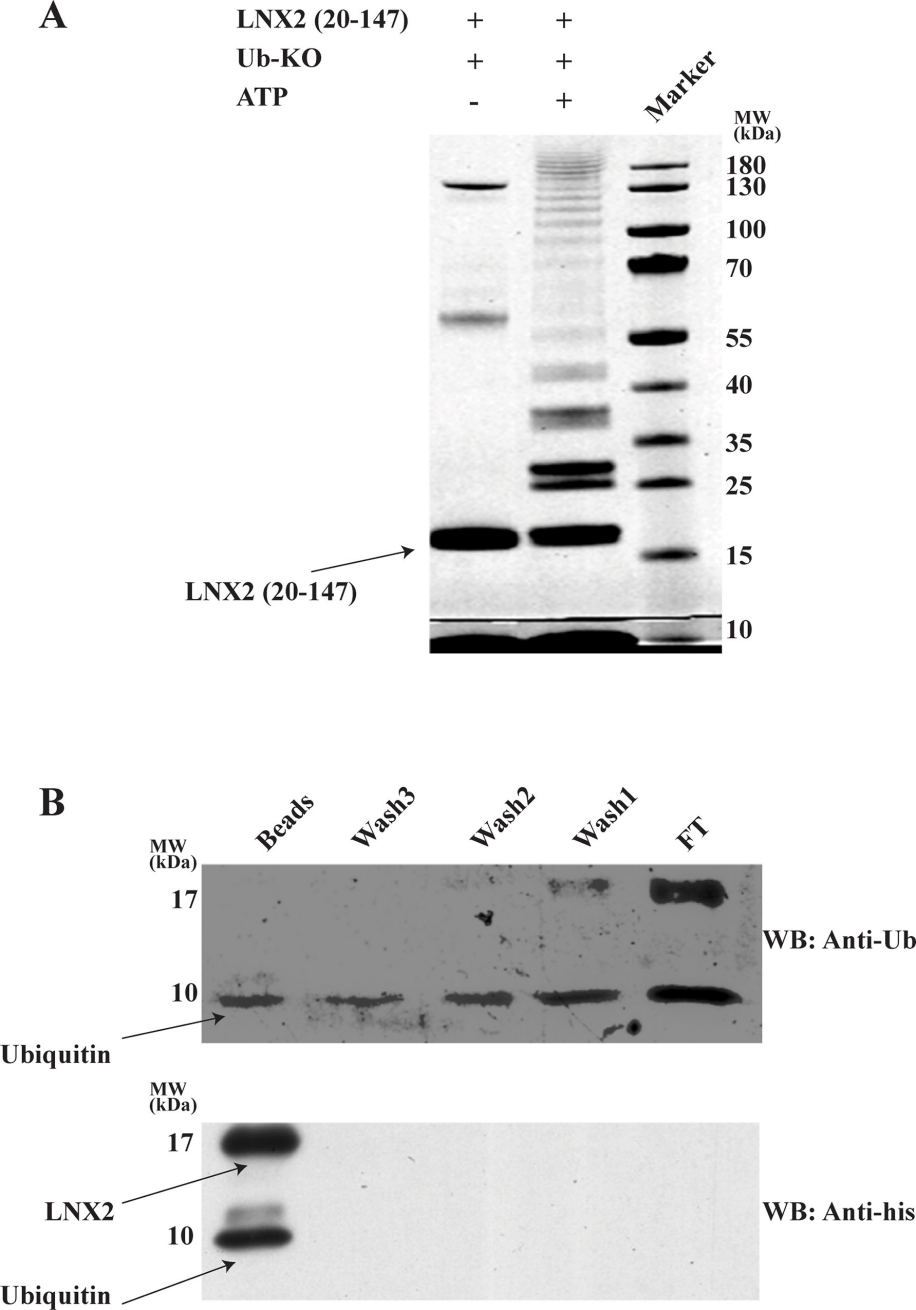
Zn-RING-Zn domain undergoes N-terminal ubiquitination **A.** *In vitro* ubiquitination was performed using E1, E2 (UbcH5b), Zn-RING-Zn domain of LNX2, Ub-KO ubiquitin (all Lys are mutated to Arg) and in the presence (+) or absence (−) of ATP. The samples were then resolved by 12.5% SDS-PAGE, followed by gel staining using coomassie brilliant blue **B.** *In vitro* ubiquitination was performed using E1, E2 (UbcH5b), ATP, 6xHistidine tagged Zn-RING-Zn domain of LNX2 and Ub-KO ubiquitin. After overnight cleavage with precission enzyme, the mixture was incubated with Ni-NTA beads followed by washing for three times. The Flow thru, washes and beads were separated by 12.5% SDS-PAGE (Supplementary Figure S8B) and subjected to western blotting. Shown is the immunoblot using anti-Ubiquitin (top) and anti-His (bottom) antibody. FT- Flow thru.

Lysine residues are well known sites for ubiquitination. The Zn-RING-Zn domain of LNX2 has ten lysine residues, of which three are in the RING domain; rest span the C-terminal region of the protein. To identify the lysine residues that are involved in autoubiquitination we performed the ubiquitination assay using a ubiquitin in which all the lysines have been mutated to arginine (Ub-KO) to prevent the formation of polyubiquitin chains. These samples were subjected to SDS-PAGE gel where multiple bands were observed corresponding to multi-monoubiquitination (Figure 8A) suggesting that all lysines are involved in autoubiquitination. We next sought to verify whether this domain undergoes N-terminal ubiquitination by adopting the protocol previously reported by others [35], with some modifications (see Methods). Using an N-terminal 6 × His-tagged Zn-RING-Zn domain (with the protease cleavage site between the His-tag and the protein) and Ub-KO, we performed a ubiquitination assay followed by protease cleavage and Ni-NTA-beads pull-down, collecting the flow-through, washes and beads for immunoblotting. The detection of a band at ~9 kDa using both anti-ubiquitin and anti-His confirmed the presence of His-tagged ubiquitin, which is only possible if Zn-RING-Zn undergoes N-terminal ubiquitination (Figure 8B and Supplementary Figure S8A). Collectively it indicates that this domain undergoes ubiquitination at its N-terminus.

## DISCUSSION

LNX2 is an E3-ubiqutin ligase known to interact with Numb and with a similar protein, Numblike [15]. In our previous work, we uncovered an E3-ligase Hakai pTyr-binding domain (HYB domain) [23], and hypothesized that LNX1 and LNX2 might harbour a similar conserved zinc-coordinating residues as shown for the HYB domain. However till date no structure-based insight into the mechanism of action of LNX2 or any of its homologues is available. To this end, here we report the minimum region responsible for the ubiquitination activity of LNX2 and identify a novel, open circle conformation Zinc finger motif at the N-terminus that is essential for its ubiquitination function. We show that although LNX2 exists as a dimer in solution, this dimerization is not required for its autoubiquitination.

In our structural homology search for the Zn-RING-Zn domain, we identified TRAF6 as the closest homologue to LNX2 (RMSD of 1.41 Å for 93 C_α_ atoms). However, the homology only extended to the RING and C-terminal Zn finger motif, with the N-terminal novel open circle Zn finger motif of LNX2 absent in TRAF6. We show that LNX2 undergoes autoubiquitination similar to that reported for TRAF6 [36]. However, TRAF6 uses a different E2-conjugating enzyme than LNX2. We speculate that the N-terminal Zn finger motif in LNX2 might play a deciding role in the selection of E2 for its activity. Moreover based on our mutational studies we have shown the essential role of this structural motif for the function of LNX2.

Further it has been reported that TRAF6 dimerization is indispensable for its autoubiquitination but LNX2 can undergo autoubiquitination as a monomer or a dimer. A similar observation has been reported for MDM2, where it was shown that RING oligomerization is not important for the protein's autoubiquitination [34].

We showed that the ubiquitin chains formed by LNX2 contain all seven possible isopeptide linkages. Formation of these polyubiquitin chains has been shown to have pathological implications, for example, in neurodegenerative diseases [37]. Similar polyubiquitin chain formation has been linked to other RING-type E3 ligases, such as MuRF1 and Mdm2, a key oncogene and negative regulator of p53 tumor suppressor [29].

**Figure 9:**
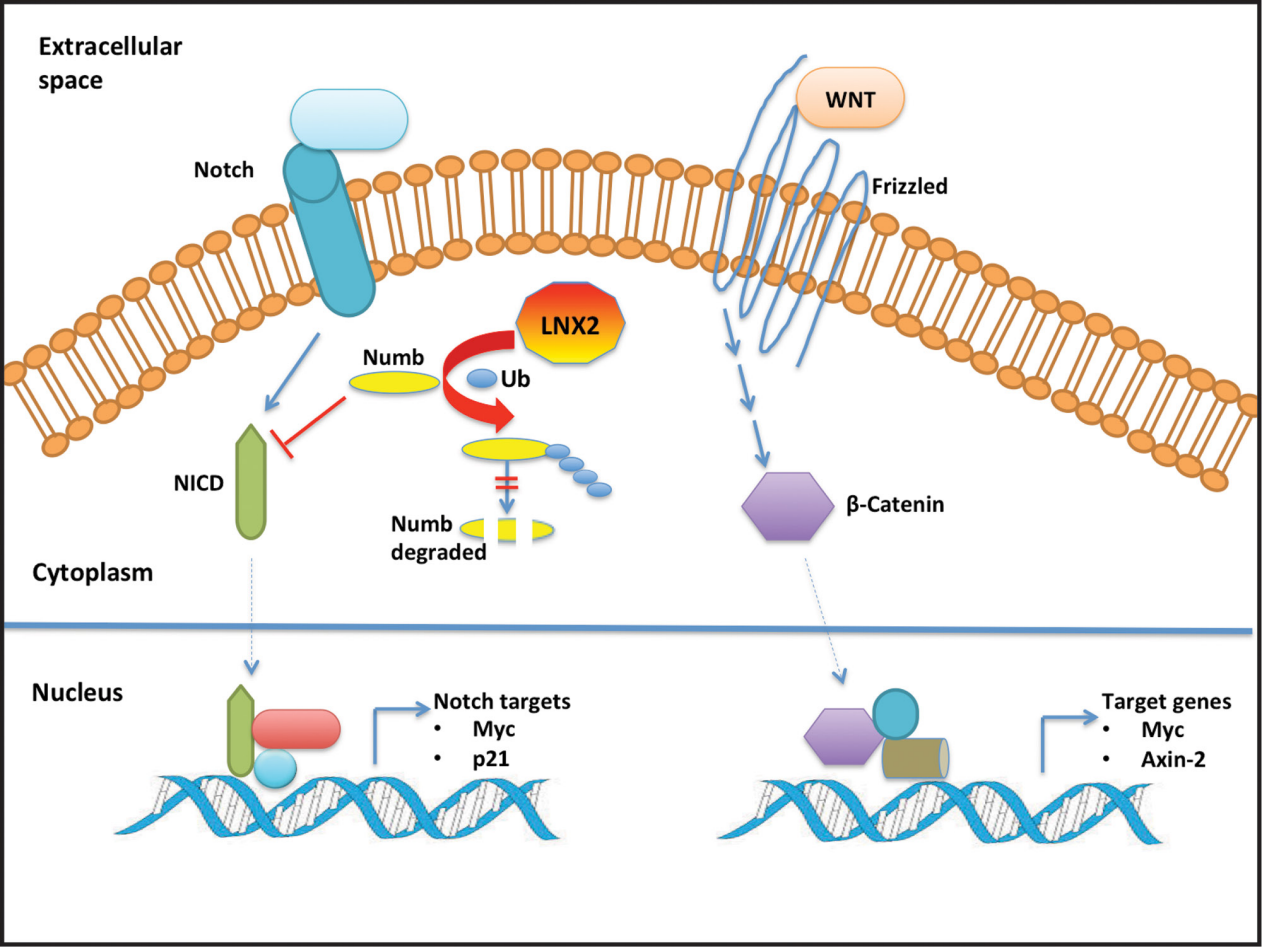
Schematic model of the cross-talk between LNX2 and WNT/NOTCH pathways For clarity, we show the effect of LNX2 on NOTCH pathway with the WNT pathway unaffected. However, NOTCH and WNT pathways have been reported to work cooperatively in tumorigenesis and hence a change in one will affect the other [41]. Based on our studies, LNX2 can bind numb and induce its ubiquitination and subsequent proteasomal degradation [44]. Numb inhibits the NOTCH pathway by degrading NICD [53]. Aberrant changes in the WNT and NOTCH pathways have been associated with cancer [54, 55]. This leads us to speculate a role for LNX2 in tumorigenesis by affecting the WNT and NOTCH pathways through the ubiquitination of Numb. Our study provides the structure of the functional machinery of LNX2 and thus a potential target for therapeutic intervention. Abbreviations: NICD, Notch intracellular domain; Ub, Ubiquitin.

As detailed earlier, LNX2 is reported to be an oncogene [19] and its silencing affects the NOTCH1 and WNT signalling pathways. Notch signalling plays a critical role in cellular proliferation, differentiation and colorectal epithelial maturation [38], and any aberrant change in this pathway could lead to cancer or tumor formation [39]. In terms of its role in WNT signalling, LNX2 modulates β-catenin and TCF7L2 [19], two key downstream effectors of WNT [40]. It has been reported that the collaborative effect of NOTCH and WNT pathways is responsible for cell proliferation and tumorigenesis [41]. Moreover LNX1 and LNX2 bind numb—an inhibitor of NOTCH pathway [42, 43]—and lead to its proteasome-dependent degradation by ubiquitination [14, 15] thereby enhancing NOTCH signalling [44, 45]. Here we showed the *in vitro* ubiquitination of Numb by LNX2. From these collective observations, we are tempted to speculate that LNX2 plays an important role in tumorigenesis by affecting WNT and NOTCH pathways through the ubiquitination of numb (Figure 9).

In conclusion, we have identified the minimum region of LNX2 (ZN-RING-Zn domain) responsible for its ubiquitination activity and studied its structure and function. We revealed the presence of a novel, open circle Zinc finger motif at the N-terminus that is indispensable for the ubiquitination function of LNX2. Furthermore, in addition to its autoubiquitination, we show that LNX2 undergoes N-terminal ubiquitination. Besides, we show the substrate E3 ligase activity of LNX2 by ubiquitination of human Numb. Our studies provide insight into the ubiquitination of LNX2 and might lead to the development of therapeutic interventions that target the Zn-RING-Zn domain.

## MATERIALS AND METHODS

### Plasmids and bacterial strain

The genes for Human LNX2 (aa20–147), full-length LNX2, Human Numb (p72) and UbcH5b were from Genscript (USA). Human Ube1 was from Addgene (Cambridge, MA) (plasmid 34965, deposited by Cynthia Wolberger) [46]. For structural and functional studies, all of the genes, with the exception of Ube1, were cloned into pGEX6P-1 (GE Healthcare, UK) or pMAL (NEB). Point mutants were generated using the proofreading HiFi DNA polymerase (Kapa Biosystems). Each sequence was verified by DNA sequencing. All clones were expressed in BL21 (DE3) bacterial strain.

### Antibodies and reagents

Monoclonal mouse anti-LNX2 antibody was from Abcam. Polyclonal rabbit anti-Ubiquitin antibody was obtained from Santa Cruz Biotechnology (SantaCruz, CA). Polyclonal rabbit Anti-Numb antibody was bought from Abcam. HRP-conjugated anti-rabbit and anti-mouse antibodies were purchased from Jackson ImmunoResearch.

### Protein purification and gel-filtration chromatography

All GST- and MBP-tagged constructs were expressed and purified using glutathione-conjugated sepharose (GE Healthcare) or Amylose sepharose (NEB), respectively. The GST- and MBP-tagged proteins were cleaved using GST-PreScission Protease (GE Healthcare) and TEV protease (Sigma), respectively. The cleaved proteins were applied to Superdex 75 or Superdex 200 size-exclusion columns (GE Healthcare) that were equilibrated using respective buffers. Ube1 was purified using Ni-NTA-conjugated sepharose (Roche). The purified protein was then applied to the Superdex 200 size-exclusion column.

### Crystallization and structure determination

Drops containing 1 μL of protein solution (7.5 mg/ml) and 1 μL of reservoir solutions were equilibrated by hanging drop vapor diffusion method at 25°C. The best crystals were grown from 0.1 M MES monohydrate pH 6.5, 12% w/v polyethylene glycol 20,000 with the protein in 10 mM Bis-Tris (pH 6.0), 100 mM NaCl, 5% glycerol and 5 mM DTT. A complete SAD data set was collected to 1.86 Å resolution at the beam line 13B1, National synchrotron radiation research center (NSRRC, Taiwan), using a Quantum-315r CCD area detector (ADSC) and processed with HKL2000 [47]. The crystals belonged to space group P 2_1_ with cell parameters as *a* = 46.97 Å, *b* = 69.39 Å, *c* = 52.25 Å and two molecules in the asymmetric unit.

All of the expected eight Zn sites of an asymmetric unit were located using the program Phenix-Autosol. The overall figure of merit was improved and over 90% of the molecule was built automatically. The remaining part of the model were built manually using COOT [48] and alternately refined by PHENIX [49]. The final model was refined to a 1.86Å resolution with an R-factor of 0.190 (R_free_= 0.232) and analysed using PROCHECK [50]. All structure-related figures were prepared using PyMOL [51].

### *In vitro* ubiquitination assays

We incubated 0.05 μM E1 (Ube1), 1 μM E2 (UbcH5b), 2 μM WT or mutant Zn-RING-Zn domain and 20 μM WT or mutant Ubiquitin at 37°C for 3 h in a reaction buffer containing 50 mM HEPES, pH 7.5, 100 mM NaCl, 10 mM MgCl_2,_ 5 mM ATP and 1 mM DTT. For substrate ubiquitination GST-Numb was added to the above reaction mixture at 2 μM concentration. The reaction was quenched using SDS loading buffer and was resolved using 12.5% SDS-PAGE. The protein bands were transferred to PVDF membranes using a Tran-Blot SD Semi-Dry Transfer Cell (Bio-Rad). The membrane was then probed with anti-ubiquitin or anti-LNX2 or anti-Numb antibody in 1:2000 dilutions followed by washing and then incubation with horseradish peroxidase (HRP)-conjugated anti-rabbit or anti-mouse secondary antibodies in a 1:10,000 dilution. The bands were visualized using ECL western blotting substrate (Pierce) as instructed by the manufacturer.

### Analytical ultracentrifugation (AUC)

The oligomeric state of the full length LNX2 was investigated by monitoring their sedimentation properties in AUC sedimentation velocity experiments. Samples (370 μl) in 50 mM HEPES pH 7.5, 100 mM NaCl and 2 mM DTT with an absorbance of 0.8 at 280 nm were used. Sedimentation velocity profiles were collected by monitoring the absorbance at 280 nm. The samples were centrifuged at 30,000 RPM at 24°C in a Beckman ProteomeLab XL-I centrifuge fitted with a four-hole AN-60 Ti rotor and double-sector aluminum centerpieces and equipped with absorbance optics. The scans were analyzed using the Sedfit program [52].

### Ni-NTA pull down assay

After the ubiquitination assay, the reaction mixture was kept for overnight cleavage with prescission enzyme. The mixture was then incubated with Ni-NTA beads in a 2 ml eppendorf at 4°C with mild rotation for 1 hr. The beads were then spun at 3381 × g for 5 mins and then the flow through was collected. The beads were then equilibrated with wash buffer for 10 mins at 4°C. This was followed up by spining the beads and collecting the wash. This step was repeated three times.

### Circular dichroism spectrometry

Far UV spectra (260–200 nm) of WT LNX2 (20-147) and its mutants (K109A, C26A, C120A) were measured using a Jasco J-810 spectropolarimeter (Jasco Europe, MI, Italy) in 20 mM Bis-Tris, 100 mM NaCl and 5 mM DTT (pH 6.0) at room temperature using a 0.1-cm path length and stoppered cuvettes. Six scans were recorded, averaged, and the baseline subtracted.

### Mass spectrometry

After ubiquitination assay, the reaction mixture was resolved on a 12,5% SDS-PAGE and stained with Coomassie Blue. The ubiquitinated species were excised from the PAGE gel and digested exhaustively with trypsin. The peptide separation was carried out on an Eksigent nanolC Ultra and ChiPLC-nanoflex (Eksigent, Dublin, CA, USA) in Trap Elute configuaration. The samples were desalted with Sep-Pak tC 18 μ Elution Plate (Waters, Miltford, MA, USA) and reconstituted with 20 μl of diluent (98% Water, 2% Acetonitrile, 0.05% Formic acid). 2 μl of the sample was loaded on a 200 μm × 0.5mm trap column and eluted on an analytical 75 μm × 150mm column. Both trap and analytical columns were made of ChromXP C18-CL, 3 μm (Eksigent, Germany). Peptides were separated by a gradient formed by 2% ACN, 0.1% FA (mobile phase A) and 98% ACN, 0.1% FA (mobile phase B): 5 to 7% of mobile phase B in 0.1min, 7 to 30% of mobile phase B in 10min, 30 to 60% of mobile phase B in 4min, 90 to 90% of mobile phase B in 5min, 90 to 5% of mobile phase B in 0.1min and 5 to 5% of mobile phase B in 10min, at a flow rate of 300. nl/min.

The MS analysis was performed on a TripleTOF 5600 system (AB SCIEX, Foster City, CA, USA) in Information Dependent Mode. MS spectra were acquired across the mass range of 400–1800 m/z in high-resolution mode (>30000) using 250 ms accumulation time per spectrum. A maximum of 20 precursors per cycle were chosen for fragmentation from each MS spectrum with 100 ms minimum accumulation time for each precursor and dynamic exclusion for 15 s. Tandem mass spectra were recorded in high sensitivity mode (resolution > 15000) with rolling collision energy on.

Peptide identification and quantification was carried on the ProteinPilot 4.5 software Revision 1656 (AB SCIEX) using the Paragon database search algorithm (4.5.0.0.) and the integrated false discovery rate (FDR) analysis function. The data were searched against a database consisting 2013_Novuniprot_sprot (total 40554 entries). The search parameters are as follows: Sample Type — Idenification; Cys Alkylation — Iodoacetamide; Digestion — trypsin; Special Factors — Gel-based ID, Ubiquitin/SUMO enrichment; Species — Homo sapines. The processing was specified as follows: ID Focus—Biological Modifications; Search Effort — Thorough; Detected Protein Threshold — 0.05 (10.0%). For FDR determination, data were searched against a concatenated database with *in silico* on-the-fly reversal for decoy sequences automatically by the software. Peptides identified with confidence interval ≥ 95% were taken into account.

## ACCESSION NUMBER

The coordinates and structure factors have been deposited at the protein Data Bank (PDB) with the accession code 5DIN.

## SUPPLEMENTARY FIGURES AND TABLE


