# Supplementary material for: Structural basis for the indispensable role of a unique zinc finger motif in LNX2 ubiquitination
Source: Oncotarget. 2015 Oct 5;6(33):34342–57. doi: 10.18632/oncotarget.5326 (PMC4741457; doi:10.18632/oncotarget.5326)
Supplement: Supplementary file 1 [file oncotarget-06-34342-s001.pdf]

SUPPLEMENTARY FIGURES AND TABLE

A

Residue	b	b+2	y	y+2
L	142.0863	71.5468	1806.99	903.9984
R	298.1874	149.5973	1665.911	833.4589
G	355.2088	178.1081	1509.809	755.4083
G	412.2303	206.6188	1452.788	726.8976
M!	559.2657	280.1365	1395.766	698.3869
Q	687.3243	344.1658	1248.731	624.8692
I	800.4083	400.7078	1120.673	560.8399
F	947.4767	474.242	1007.588	504.2978
V	1046.545	523.7762	860.52	430.7636
K*	1288.683	644.8452	761.4516	381.2294
T	1389.731	695.369	519.3137	260.1605
L	1502.815	751.911	418.266	209.6366
T	1603.863	802.4349	305.1819	153.0946
G	1660.884	830.9456	204.1343	102.5708
K	1788.979	894.9931	147.1128	74.06

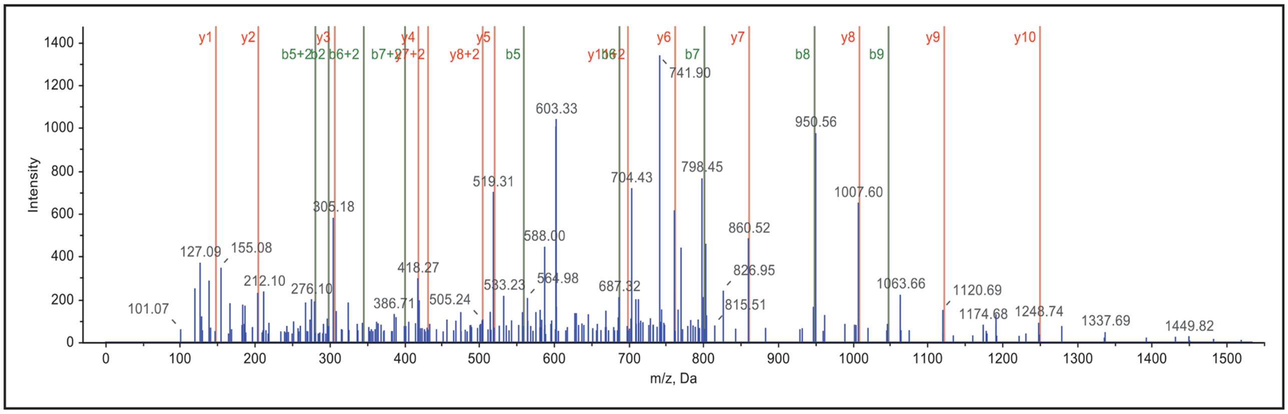

Ubiquitination at Lys6: LRGGM!QIFVK*TLGK

Supplementary Figure S1: Spectrum of peptides obtained after tryptic digest of ubiquitinated species. After the ubiquitination assay, the mixture is resolved on 12.5% SDS-PAGE gel and the ubiquitinated species were excised, digested with trypsin and analysed by mass spectrometery. Each spectrum indicates the type of isopeptide linkage formed. K*- lysine residue modified by di-peptide (-GG). K**- lysine residue modified by (-LRGG) peptide due to misclevage. M!- oxidized methionine.

B

Residue	b	b+2	y	y+2
T	102.055	51.5311	2444.288	1222.648
L	215.139	108.0731	2343.24	1172.124
T	316.1867	158.597	2230.156	1115.582
G	373.2082	187.1077	2129.109	1065.058
K**	884.5312	442.7693	2072.087	1036.547
T	985.5789	493.2931	1560.764	780.8856
I	1098.663	549.8351	1459.716	730.3618
T	1199.711	600.359	1346.632	673.8197
L	1312.795	656.901	1245.585	623.2959
E	1441.837	721.4223	1132.5	566.7539
V	1540.906	770.9565	1003.458	502.2326
E	1669.948	835.4778	904.3894	452.6984
P	1767.001	884.0042	775.3468	388.1771
S	1854.033	927.5202	678.2941	339.6507
D	1969.06	985.0337	591.262	296.1347
T	2070.108	1035.558	476.2351	238.6212
I	2183.192	1092.1	375.1874	188.0974
E	2312.234	1156.621	262.1034	131.5553
N	2426.277	1213.642	133.0608	67.034

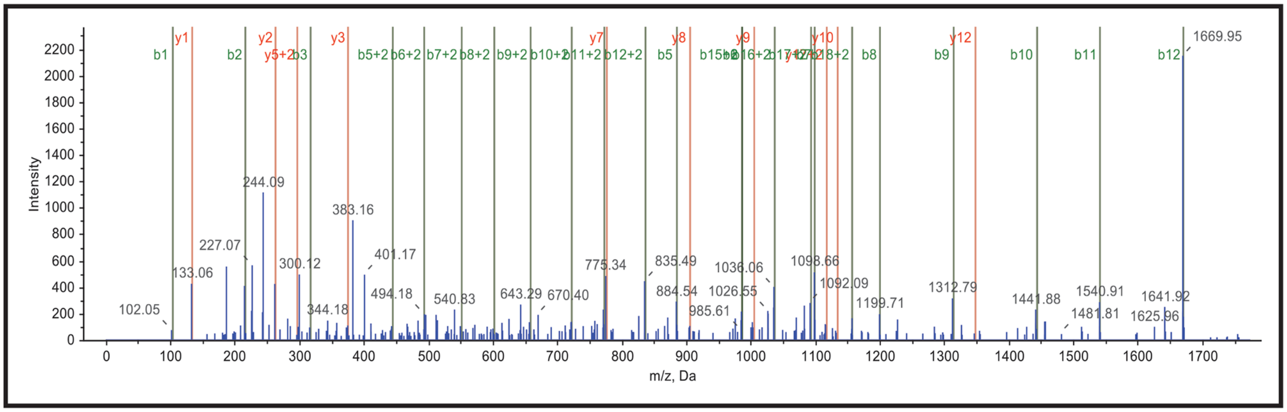

Ubiquitination at Lys 11: TLTGK**TITLEVEPSDTIEN

C

Residue	b	b+2	y	y+2
T	102.055	51.5311	2530.325	1265.666
L	215.139	108.0731	2429.277	1215.142
T	316.1867	158.597	2316.193	1158.6
G	373.2082	187.1077	2215.145	1108.076
K*	615.3461	308.1767	2158.124	1079.566
T	716.3937	358.7005	1915.986	958.4966
I	829.4778	415.2425	1814.938	907.9727
T	930.5255	465.7664	1701.854	851.4307
L	1043.61	522.3084	1600.807	800.9069
E	1172.652	586.8297	1487.722	744.3648
V	1271.721	636.3639	1358.68	679.8435
E	1400.763	700.8852	1259.611	630.3093
P	1497.816	749.4116	1130.569	565.788
S	1584.848	792.9276	1033.516	517.2617
D	1699.875	850.4411	946.484	473.7456
T	1800.923	900.9649	831.4571	416.2322
I	1914.007	957.5069	730.4094	365.7083
E	2057.065	1029.036	617.3253	309.1663
N	2171.108	1086.058	474.2671	237.6372
V	2270.176	1135.592	360.2241	180.6157
K*	2512.314	1256.661	261.1557	131.0815

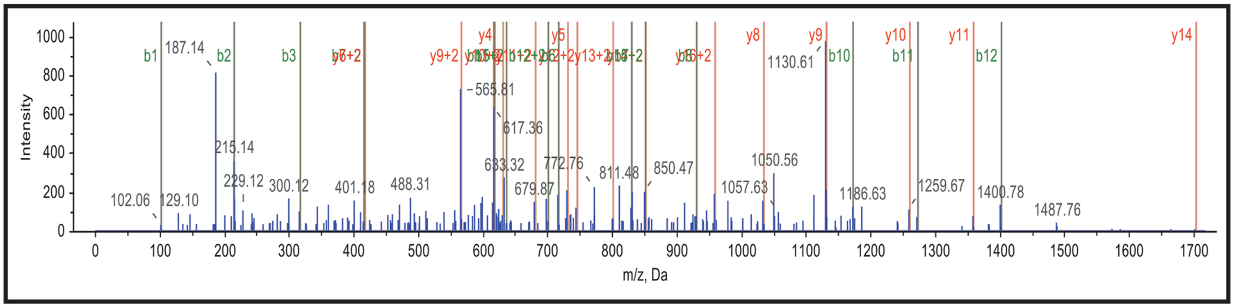

Ubiquitination at Lys11 and Lys27: TLTGK*TITLEVEPSDTI-ENNK*

D

Residue	b	b+2	y	y+2
A	115.0502	58.0287	1879.962	940.4847
K*	357.1881	179.0977	1765.919	883.4632
I	470.2722	235.6397	1523.781	762.3943
Q	598.3307	299.669	1410.697	705.8522
D	713.3577	357.1825	1282.639	641.8229
K	841.4526	421.23	1167.612	584.3095
E	970.4952	485.7513	1039.517	520.262
G	1027.517	514.262	910.4741	455.7407
I	1140.601	570.804	853.4526	427.23
P	1237.654	619.3304	740.3686	370.6879
P	1334.706	667.8568	643.3158	322.1615
D	1449.733	725.3703	546.2631	273.6352
Q	1577.792	789.3995	431.2361	216.1217
Q	1705.85	853.4288	303.1775	152.0924
R	1861.952	931.4794	175.119	88.0631

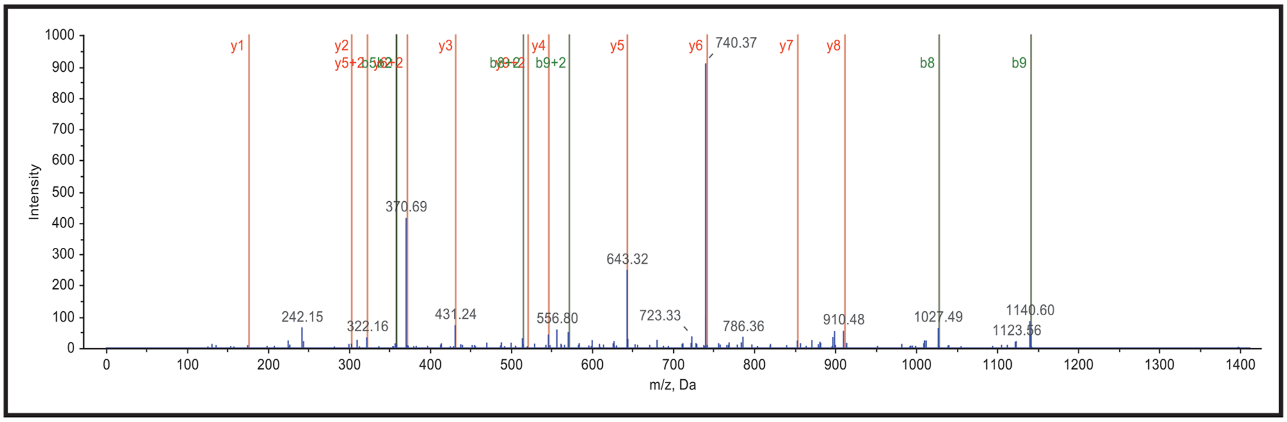

Ubiquitination at Lys29: AK*IQDKEAGIPPDQQR

E

Residue	b	b+2	y	y+2
I	114.0913	57.5493	1637.824	819.4157
Q	242.1499	121.5786	1524.74	762.8737
D	357.1769	179.0921	1396.682	698.8444
K*	599.3148	300.161	1281.655	641.3309
E	728.3573	364.6823	1039.517	520.262
G	785.3788	393.193	910.4741	455.7407
I	898.4629	449.7351	853.4526	427.23
P	995.5156	498.2615	740.3686	370.6879
P	1092.568	546.7878	643.3158	322.1615
D	1207.595	604.3013	546.2631	273.6352
Q	1335.654	668.3306	431.2361	216.1217
Q	1463.713	732.3599	303.1775	152.0924
R	1619.814	810.4104	175.119	88.0631

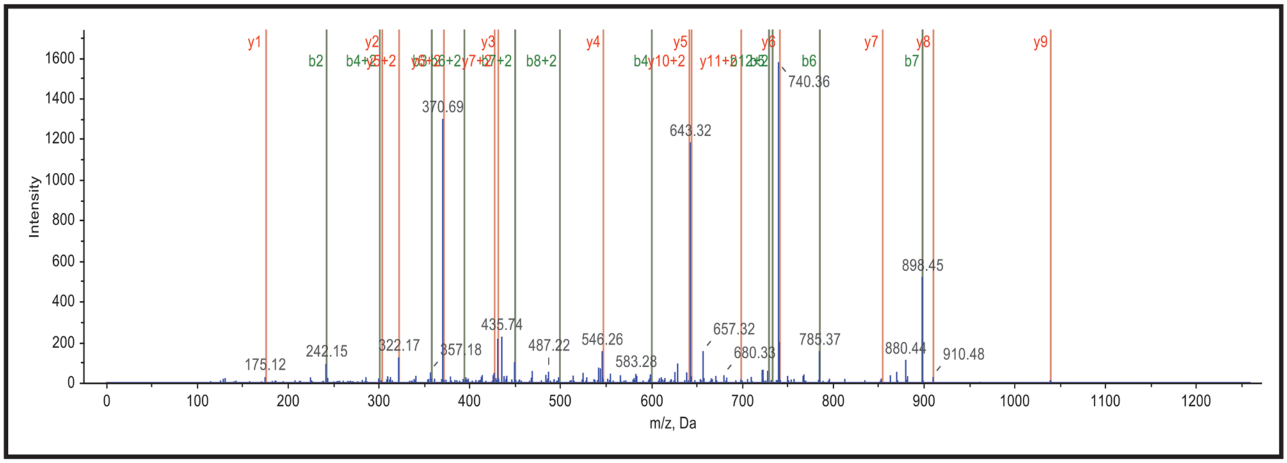

Ubiquitination at Lys33: IQDK*EGIPPDQQR

F

Residue	b	b+2	y	y+2
L	242.1863	121.5968	1588.881	794.9439
I	355.2704	178.1388	1347.702	674.3544
F	502.3388	251.673	1234.618	617.8124
A	573.3759	287.1916	1087.549	544.2782
G	630.3974	315.7023	1016.512	508.7596
K*	872.5352	436.7713	959.4905	480.2489
Q	1000.594	500.8006	717.3526	359.1799
L	1113.678	557.3426	589.294	295.1506
E	1242.721	621.8639	476.21	238.6086
D	1357.747	679.3774	347.1674	174.0873
G	1414.769	707.8881	232.1404	116.5738
R	1570.87	785.9386	175.119	88.0631

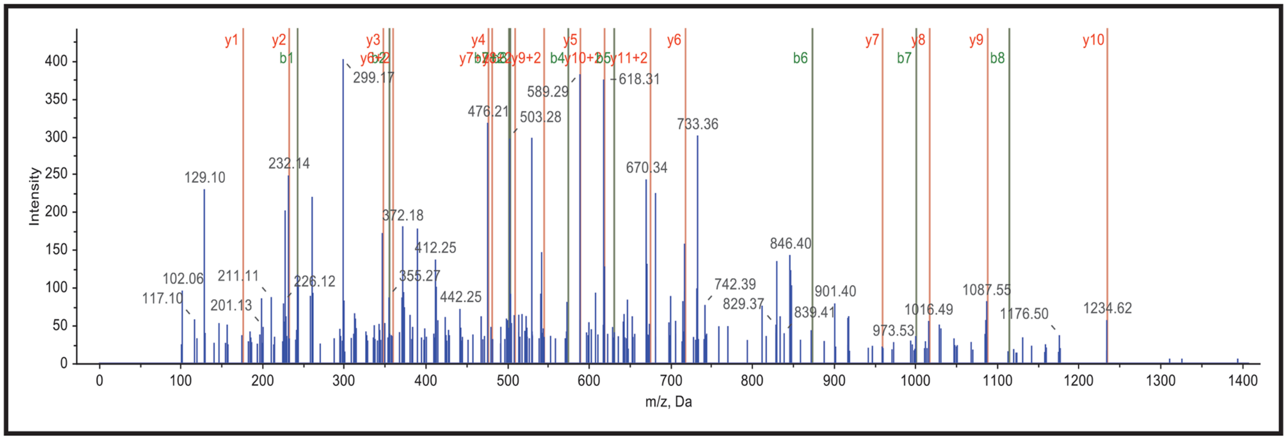

Ubiquitination at Lys48: LIFAGK*QLEDGR

G

Residue	b	b+2	y	y+2
T	102.055	51.5311	2276.188	1138.598
L	215.139	108.0731	2175.14	1088.074
S	302.171	151.5892	2062.056	1031.532
D	417.198	209.1026	1975.024	988.0158
Y	580.2613	290.6343	1859.997	930.5023
N	694.3042	347.6558	1696.934	848.9707
I	807.3883	404.1978	1582.891	791.9492
Q	935.4469	468.2271	1469.807	735.4072
K*	1177.585	589.296	1341.749	671.3779
E	1306.627	653.8173	1099.611	550.3089
S	1393.659	697.3333	970.568	485.7876
T	1494.707	747.8572	883.536	442.2716
L	1607.791	804.3992	782.4883	391.7478
H	1744.85	872.9287	669.4042	335.2058
L	1857.934	929.4707	532.3453	266.6763
V	1957.003	979.0049	419.2613	210.1343
L	2070.087	1035.547	320.1928	160.6001
R	2258.178	1129.592	207.1088	104.058

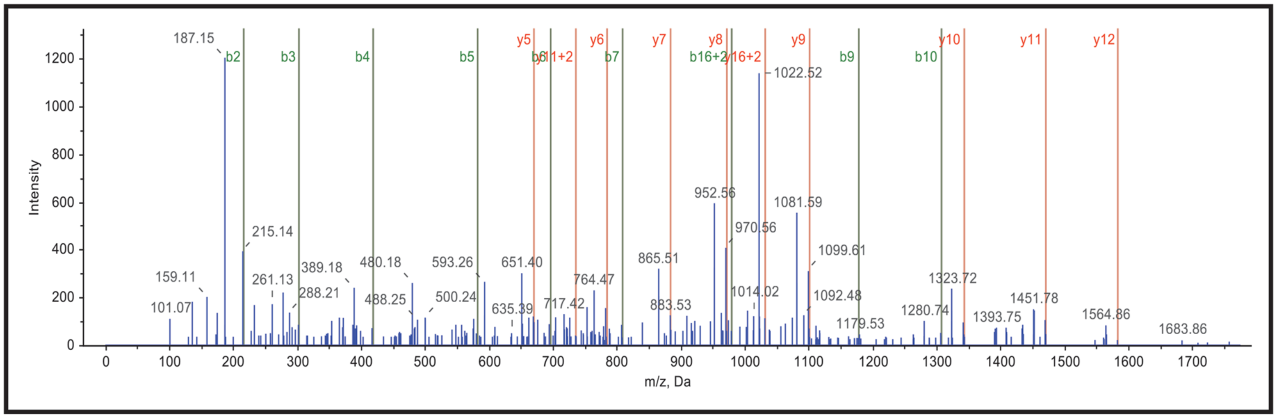

Ubiquitination of Lys63: TLSDYNIQK*ESTLHLVLR

H

Residue	b	b+2	y	y+2
L	142.0863	71.5468	3689.942	1845.475
R	298.1874	149.5973	3548.863	1774.935
G	355.2088	178.1081	3392.762	1696.885
G	412.2303	206.6188	3335.74	1668.374
M!	559.2657	280.1365	3278.719	1639.863
Q	687.3243	344.1658	3131.683	1566.345
I	800.4083	400.7078	3003.625	1502.316
F	947.4767	474.242	2890.541	1445.774
V	1046.545	523.7762	2743.472	1372.24
K*	1288.683	644.8452	2644.404	1322.706
T	1389.731	695.369	2402.266	1201.637
L	1502.815	751.911	2301.218	1151.113
T	1603.863	802.4349	2188.134	1094.571
G	1660.884	830.9456	2087.087	1044.047
K*	1903.022	952.0146	2030.065	1015.536
T	2004.07	1002.538	1787.927	894.4673
I	2117.154	1059.08	1686.88	843.9434
T	2218.201	1109.604	1573.796	787.4014
L	2331.285	1166.146	1472.748	736.8776
E	2460.328	1230.668	1359.664	680.3355
V	2559.396	1280.202	1230.621	615.8142
E	2688.439	1344.723	1131.553	566.28
P	2785.492	1393.25	1002.51	501.7587
S	2872.524	1436.766	905.4575	453.2324
D	2987.551	1494.279	818.4254	409.7164
T	3088.598	1544.803	703.3985	352.2029
I	3201.682	1601.345	602.3508	301.679
E	3330.725	1665.866	489.2667	245.137
N	3444.768	1722.888	360.2241	180.6157
V	3543.836	1772.422	246.1812	123.5942
K	3671.931	1836.469	147.1128	74.06

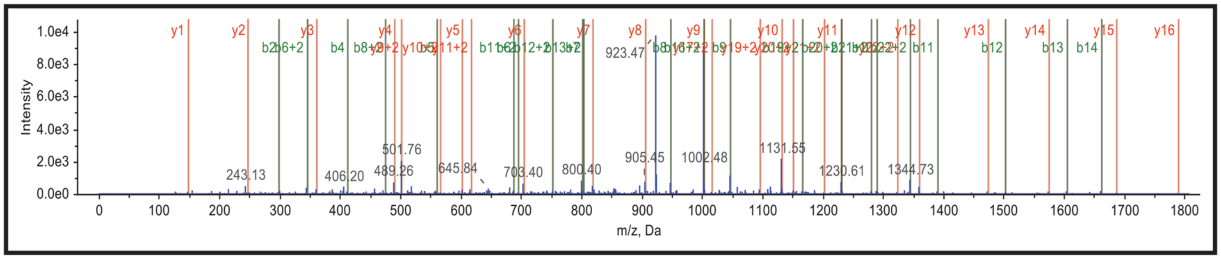

Ubiquitination at Lys6 and Lys11: LRGGM!QIFVK*TLTGK*-TITLEVEPSDTIENVK

I

Residue	b	b+2	y	y+2
A	72.0444	36.5258	2220.184	1110.596
K**	583.3675	292.1874	2149.147	1075.077
I	696.4515	348.7294	1637.824	819.4157
Q	824.5101	412.7587	1524.74	762.8737
D	939.537	470.2722	1396.682	698.8444
K*	1181.675	591.3411	1281.655	641.3309
E	1310.718	655.8624	1039.517	520.262
G	1367.739	684.3731	910.4741	455.7407
I	1480.823	740.9152	853.4526	427.23
P	1577.876	789.4415	740.3686	370.6879
P	1674.929	837.9679	643.3158	322.1615
D	1789.956	895.4814	546.2631	273.6352
Q	1918.014	959.5107	431.2361	216.1217
Q	2046.073	1023.54	303.1775	152.0924
R	2202.174	1101.591	175.119	88.0631

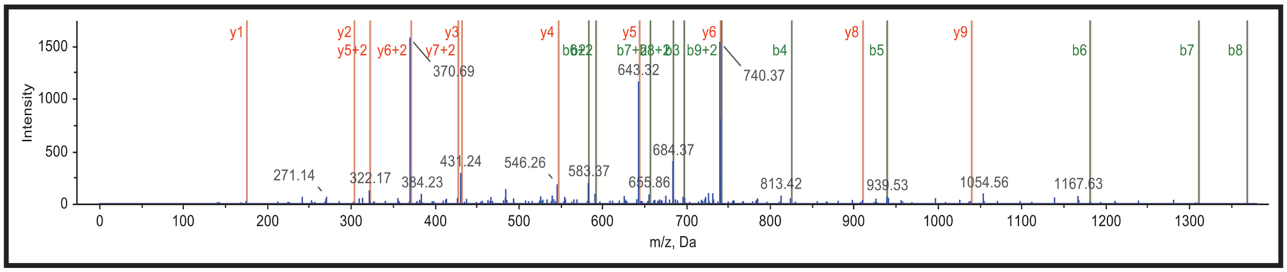

Ubiquitination at Lys29 and Lys33: AKIQDK*EGIPPDQQR**

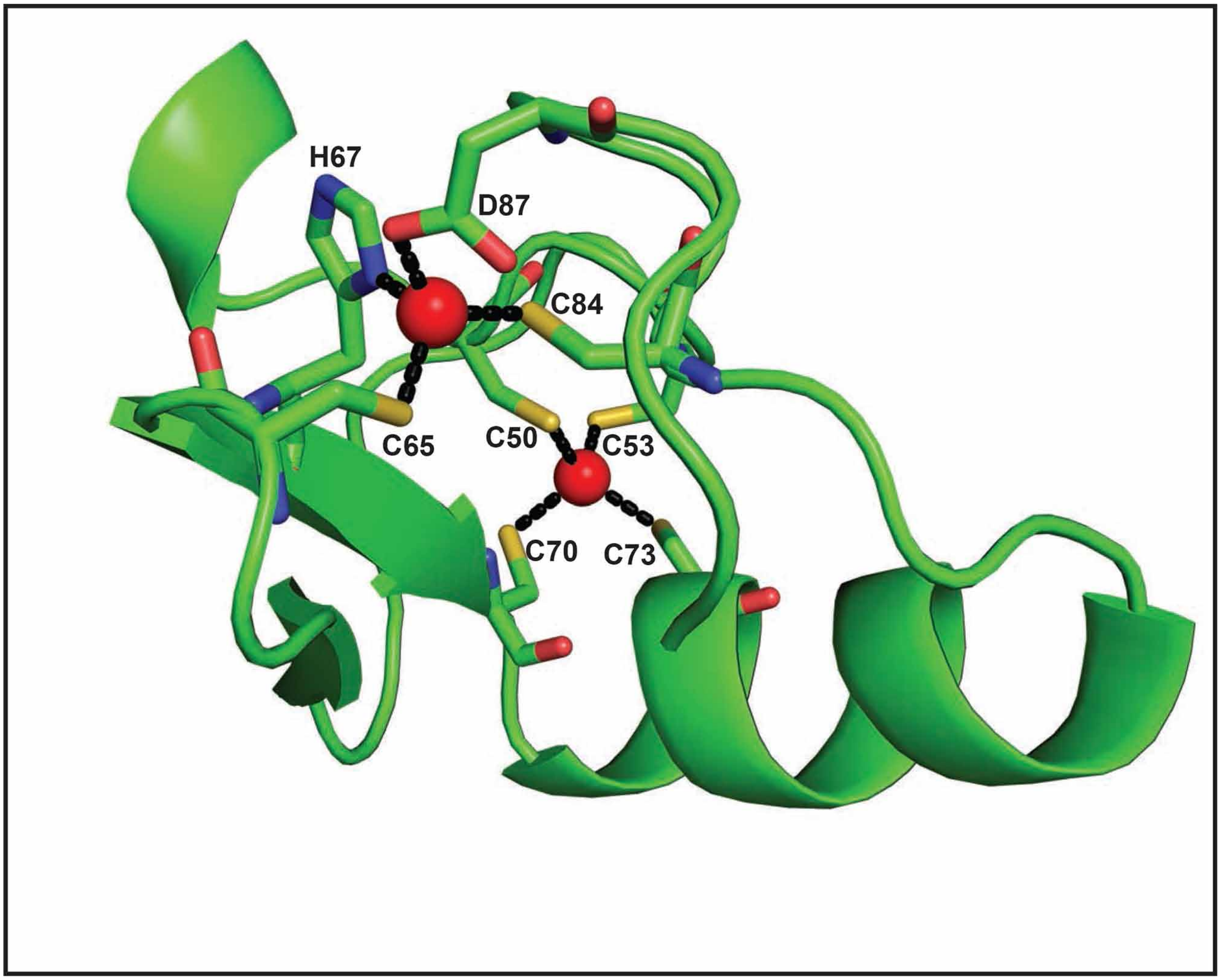

Supplementary Figure S2: Strucure of RING. The structure of the RING domain of LNX2 with zinc coordination residues and zinc ions (red spheres) for one of the monomers. Unlike the Cys4HisCys3 "cross-brace" formation, one of the cysteine residues is replaced by an aspartic acid residue (Asp87) as shown here.

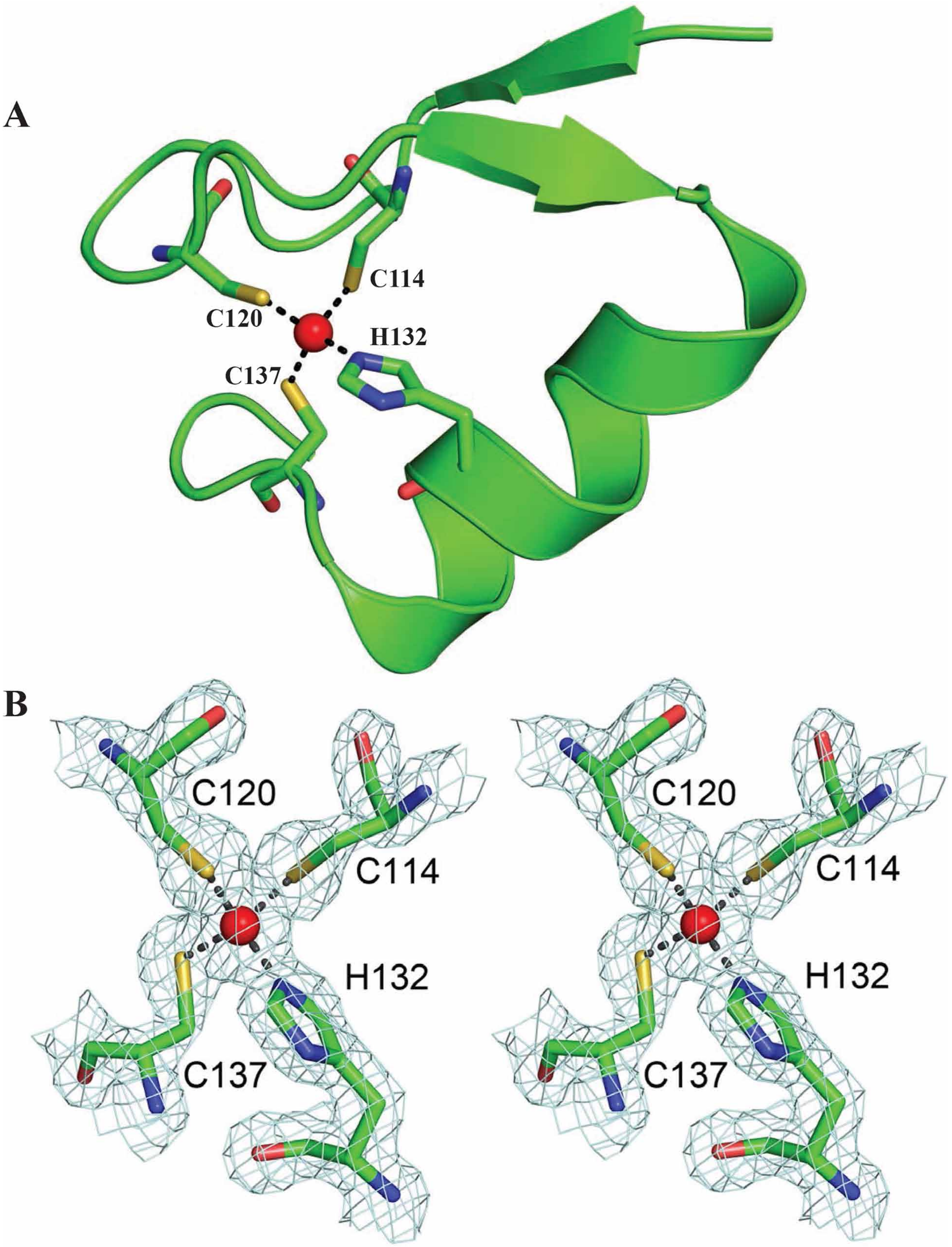

Supplementary Figure S3: C-terminal Zn finger motif in Zn-RING-Zn domain. A. Structure of the C-Terminal Zn finger motif in Zn-RING-Zn domain with zinc coordination residues and Zinc ion (red sphere). **B.** Stereo view of the zinc coordination residues and zinc ion in (A) with the final 2Fo-Fc electron density map contoured at 1.6 σ.

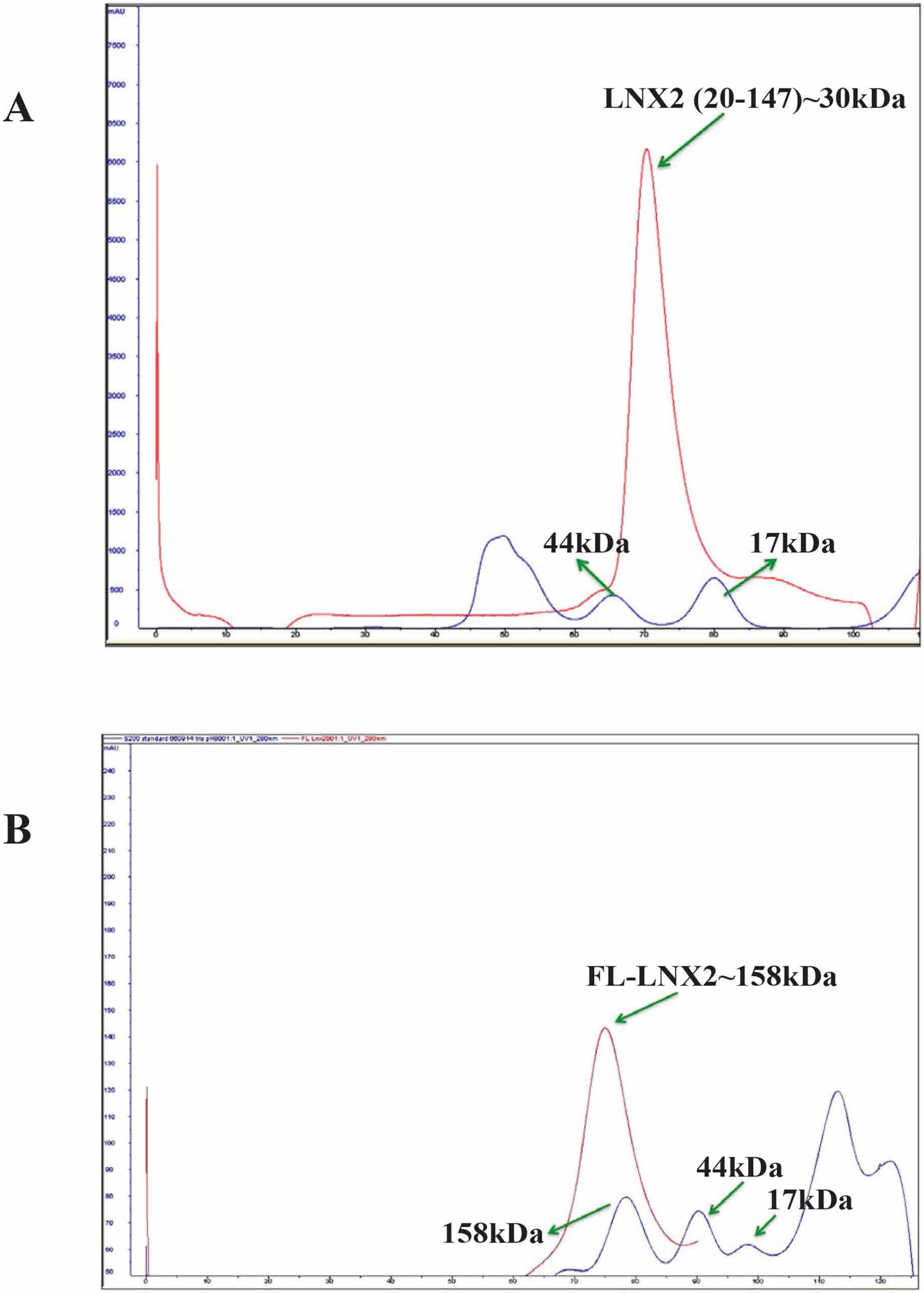

Supplementary Figure S4: Gel filteration profiles of Zn-RING-ZN domain and FL-LNX2 respectively. Zn-RING-ZN domain and FL-LNX2 elute as a single peak at an elution volume corresponding to molecular mass of approximately 30 kDa **A.** and 158 kDa **B.** respectively. The molecular mass standard is shown in blue.

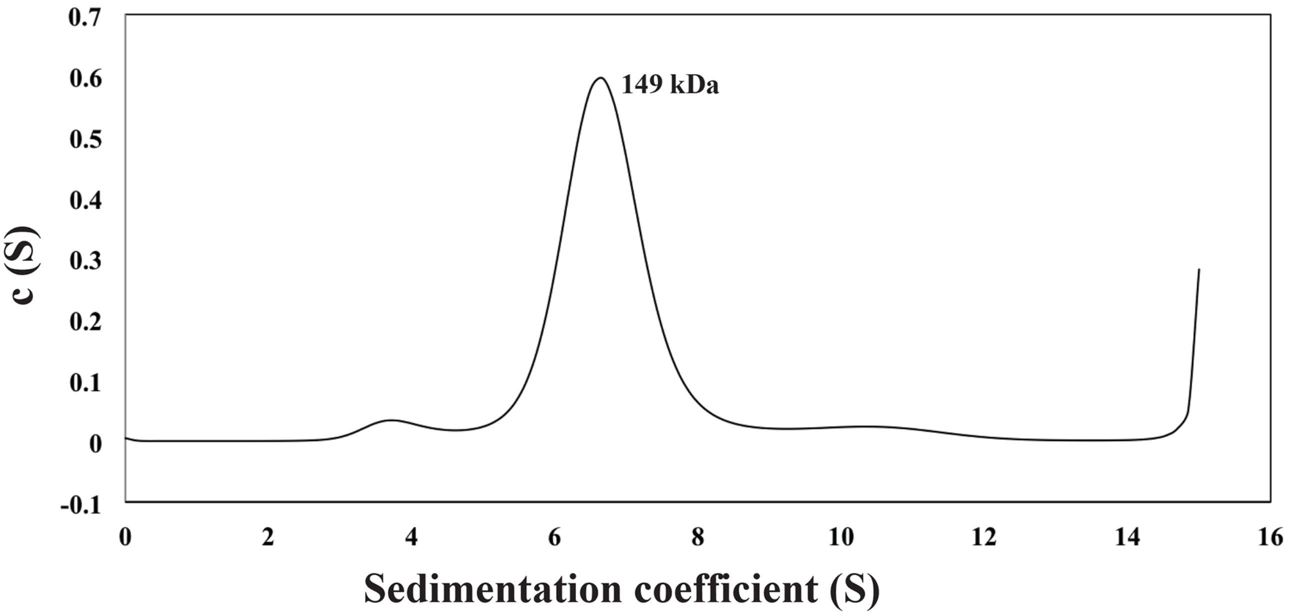

Supplementary Figure S5: Analytical Centrifugation of FL-LNX2. AUC analysis of FL-LNX2 showed the presence of dimers in the solution.

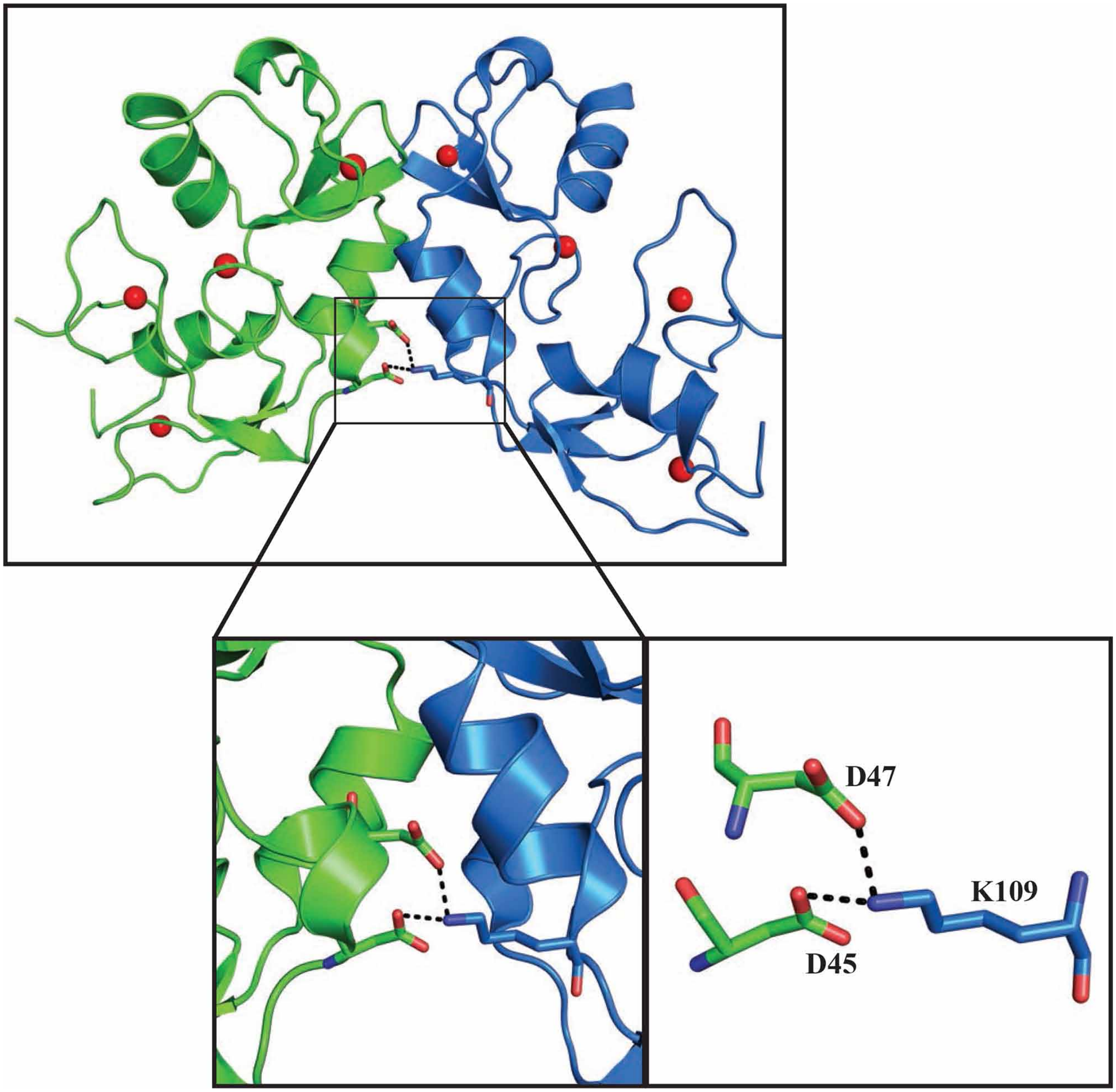

Supplementary Figure S6: Residues responsible for the dimerization of Zn-RING-Zn domain. Lys109 of one chain makes hydrogen bond contact with Asp45 and Asp47 of other chain.

A

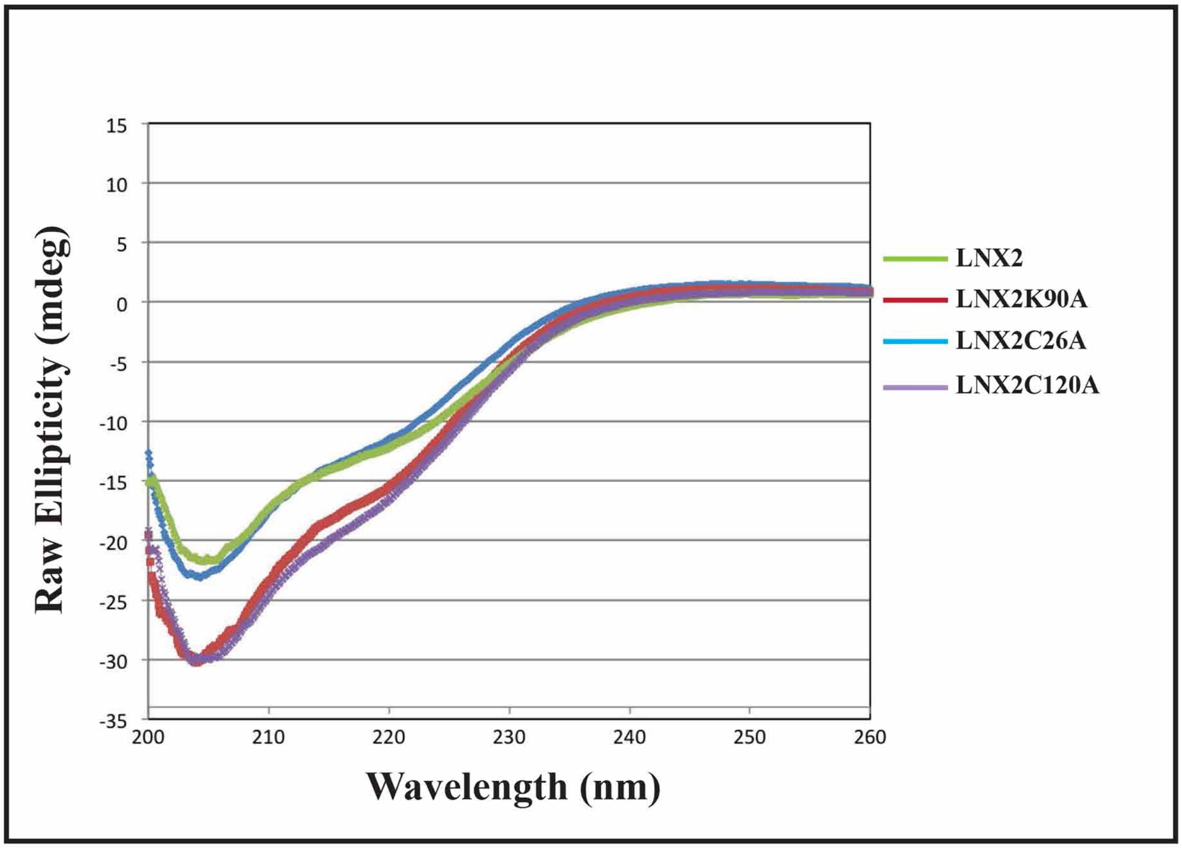

B

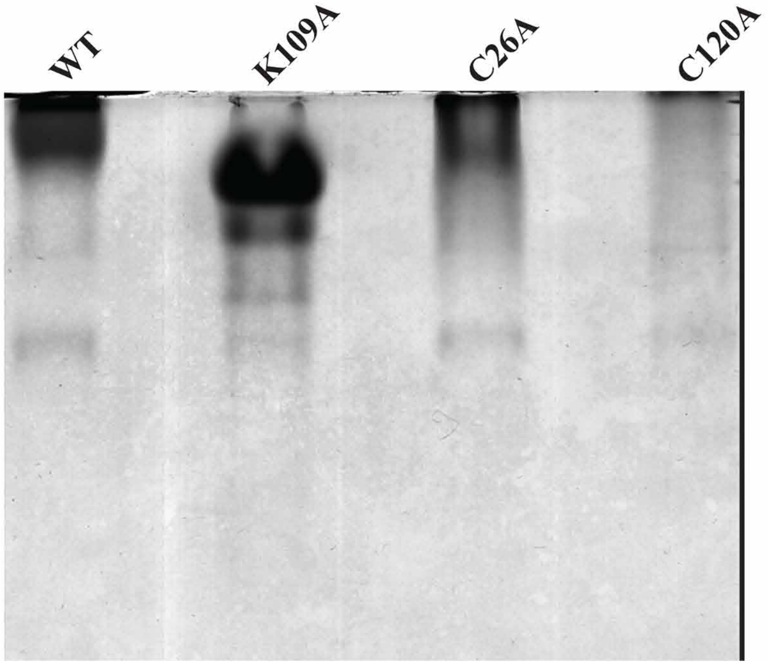

Supplementary Figure S7: Secondary structure of dimer and Zn finger motifs disruptive mutants of Zn-RING-Zn domain respectively. A. Circular dichroism analysis shows that the dimeric and two zinc finger disruptive mutants of Zn-RING-Zn respectively maintained a well-defined secondary structure, comparable to that of the wild type. **B.** Native gel analysis of the mutants used in (A) shows the change in oligomerization state of K109A mutant compared to wild type.

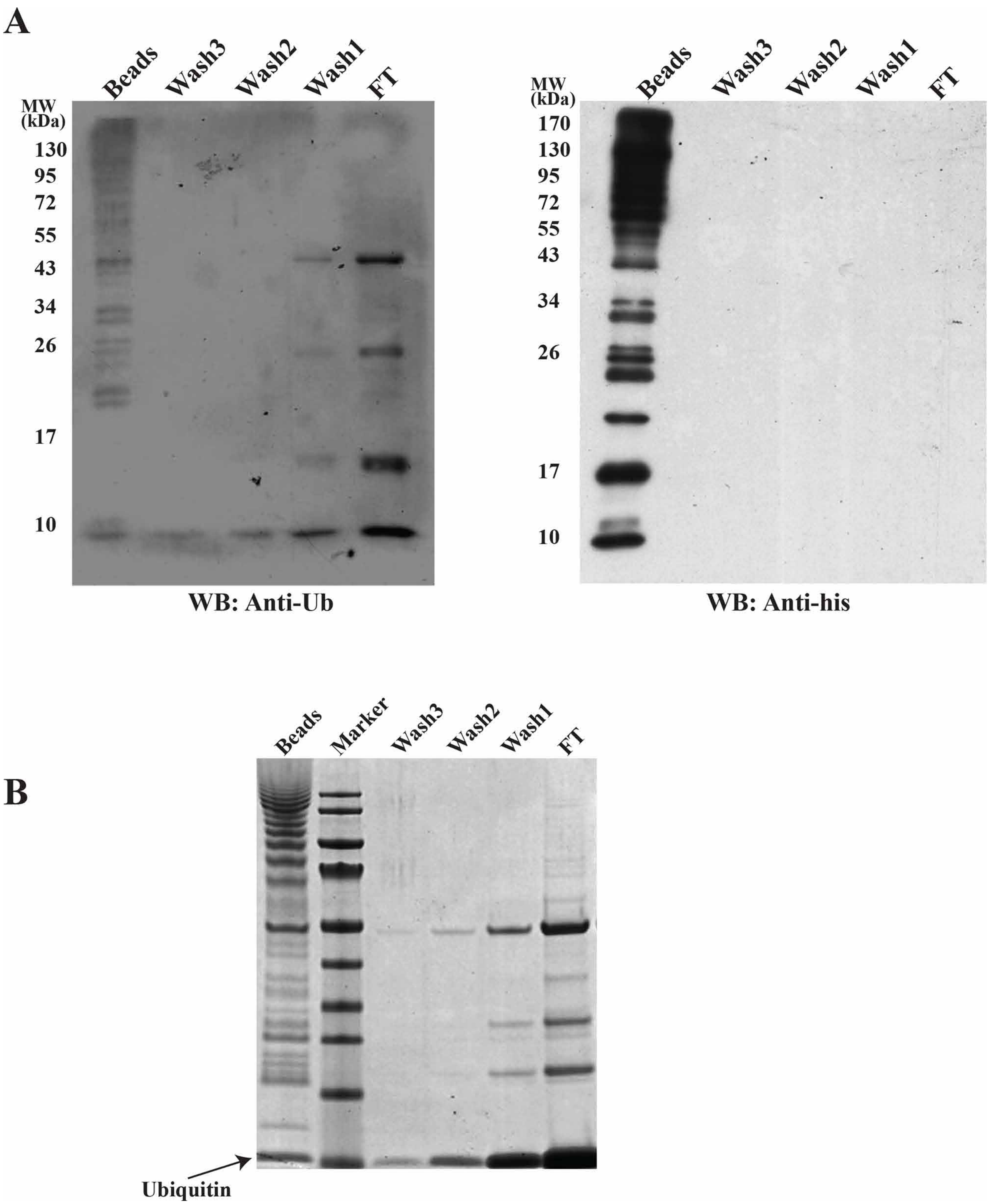

Supplementary Figure S8: Full blots of the partial western blots shown in the main text. Figure 8B. **B.** SDS-PAGE result for the corresponding blot used in **A.**

Supplementary Table S1: Crystallographic data and refinement statistics

	Zinc-SAD
**DATA COLLECTION**	
Space group	P 1 21 1
Cell Dimensions<br>a, b, c (Å)<br>α, β, γ (°)	 46.97 69.39 52.25<br>90 115.41 90
Wavelength (Å)	1.282
Resolution range (Å)	24.24 – 1.86 (1.93 – 1.86)
Total reflections	179976
Unique reflections	25098 (2367)
R-sym[a]	0.1 (0.307)
Multiplicity	7.2 (6.3)
Completeness (%)	98.92 (93.78)
Mean I/sigma(I)	9.91 (5.01)
**REFINEMENT**	
Wilson B-factor	17.84
R-factor[b]	0.190 (0.222)
R-free[c]	0.232 (0.272)
Number of atoms	2260
macromolecules	1997
ligands	20
water	243
Protein residues	244
RMS(bonds)	0.007
RMS(angles)	1.10
Ramachandran favored (%)	99
Ramachandran outliers (%)	0
Clashscore	3.01
Average B-factor	22.60
macromolecules	22.10
solvent	26.50

Statistics for the highest-resolution shell are shown in parentheses.
[a]Rsym =Σ|Ii − <I>|/Σ|Ii|, where I is the intensity of the i-th measurement, and <I> is the mean intensity for that reflection.
[b]Rwork = Σ||Fobs|−|Fcalc||/Σ|Fobs|, where Fcalc and Fobs are the calculated and observed structure factor amplitudes, respectively.
[c]Rfree = as for Rwork, but was calculated using 10% of data excluded from refinement.
